# Asphaltene biotransformation by a novel enzyme thiol peroxidase from *Micrococcus* sp. IITD107

**DOI:** 10.1128/aem.00151-25

**Published:** 2025-08-21

**Authors:** Nidhi Patil, Preeti Srivastava

**Affiliations:** 1 Department of Biochemical Engineering & Biotechnology, Indian Institute of Technology Delhi 28817, New Delhi, India; Kyoto University, Kyoto, Japan

**Keywords:** porous carbon, packed bed column, immobilized enzyme, crude oil

## Abstract

Asphaltenes are known to cause high density and viscosity in heavy crude oil. The gene for thiol peroxidase was found to be present in five members of the nine-membered bacterial consortium able to biotransform about 75% of asphaltenes. Here, we report cloning, expression, purification, and detailed characterization of a thiol peroxidase enzyme from one of the consortium members, *Micrococcus* sp. IITD107. Asphaltene was treated with the crude cell lysate obtained after overexpression of thiol peroxidase from heterologous host *Escherichia coli* as well as with purified enzyme. A significant reduction in the major peaks obtained by gas chromatography mass spectrometry (GC-MS) of asphaltene was observed, corresponding to small polyaromatic hydrocarbons such as benzaldehyde, phenol-methylethylidene, benzenepropionic acid, and linear aliphatic chains such as heptadecane, tetradecane, octadecane, etc. Changes in the structure of asphaltene were also observed in the Fourier transform infrared spectroscopy and nuclear magnetic resonance spectra. The elemental analysis determined around 60% reduction in sulfur and 69% reduction in nitrogen. A decrease in aromaticity of asphaltene was also observed. Scanning electron microscopy imaging of the treated asphaltene fraction captured during the course of biotransformation revealed formation of pores in the structure. This is the first report demonstrating the use of thiol peroxidase for asphaltene biotransformation. The enzyme can also be used for the biological synthesis of porous carbon, which has not been reported to date.

**IMPORTANCE:**

Heavy crude oil is abundant but contains asphaltene, which results in its high density and viscosity, making it unsuitable for commercial application. The removal of asphaltene leads to a reduction in heaviness of the oil and renders it light and suitable for commercialization. Asphaltene is a complex and large polyaromatic hydrocarbon consisting of several heteroatoms. The use of enzymatic biotransformation of asphaltene aids in breaking down the complex molecule into smaller moieties without affecting the calorific value. This study helps in identifying a novel enzyme thiol peroxidase for biotransformation of asphaltene and valorization of asphaltene to synthesize porous carbon.

## INTRODUCTION

Asphaltenes are high-molecular-weight hydrocarbons that are the most recalcitrant fraction of crude oil (1). They are made up of multiple aromatic rings and alkane chains and also have the presence of several heteroatoms such as S, N, O, Zn, Ni, V, and Fe. Asphaltenes are responsible for adding up to the viscosity and density of crude oil. Several physical and chemical methods have been suggested to reduce the amount of asphaltene in heavy crude oil; however, they face limitations of high energy consumption and cost. Biological degradation of asphaltene has been demonstrated using single microorganisms as well as microbial consortiums (2–6).

The use of microorganisms faces limitations of growth due to nutrient unavailability. This can be overcome by the use of enzymes. Enzymes show the ease of implementation, higher rate of action, increased specificity, and increased selectivity while carrying out the conversion of complex structures to simple compounds (7). The first-ever report of enzymatic asphaltene biotransformation was published in 1993 by Fedorak, in which the extracellular enzyme chloroperoxidase, produced by a fungus, *Caldariomyces fumago*, was found to act on the components of asphaltene and alter them. About 20% of the total V and Ni content was reported to be reduced in the asphaltene fraction due to the action of this enzyme (8). Mogollon in 1998 reported a 53% reduction in Ni and 27% reduction in V from asphaltene due to the action of the chloroperoxidase enzyme (9). In the presence of heavy crude oil, the amount of ligninolytic enzymes such as laccase, manganese peroxidase, and lignin peroxidase was found to be upregulated in the fungus *Daedaleopsis* sp., which was found to degrade 88.7% of the asphaltene fraction in the oil, indicating their role in the process of biodegradation of the target compound (5).

Several peroxidases have been known to aid in the removal of contaminants such as polyaromatic hydrocarbons (PAHs), phenolic compounds, synthetic dyes, herbicides, pharmaceutical wastes, insecticides, and others (10, 11). Lignin peroxidases and manganese peroxidases, along with several other enzymes such as laccases, oxidases, quinone reductase, and esterases, have been found to degrade certain polyaromatic hydrocarbons (12, 13). The enzymes reduce hydroperoxides by an enzyme substitution mechanism. Thiol peroxidases belong to the oxidoreductase class of enzyme and are around 20 kDa–30 kDa in size (14). The enzyme is usually produced by bacteria as a stress response element (15). Thiol peroxidases carry out the catalysis reaction using thiols. These enzymes do not need any cofactor or prosthetic group to show their activity and hence act as naked protein. These proteins mostly have a cysteine residue at their active sites that undergoes redox shuttling while carrying out their catalytic activity (16). In mammals, multiple thiol peroxidase isoenzymes have been found to combat oxidative stress in different organelles of the cell (17). Apart from combating oxidative stress, thiol peroxidase has been reported to have a dual function of being involved in gene signaling as well (18). However, no reports are available on the action of thiol peroxidase on aromatic hydrocarbon degradation or its mechanism.

Previously, we reported that a nine-membered microbial consortium consisting of *Arthrobacter* sp. IITD100*, Arthrobacter* sp. IITD101, *Rhodococcus* sp. IITD102*, Barrientosiimonas* sp. IITD103*, Lysinibacillus* sp. IITD104*, Sporosarcina* sp. IITD105*, Bacillus* sp. IITD106*, Micrococcus* sp. IITD107, and *Paenibacillus* sp. IITD108 was capable of biotransforming up to 75% of asphaltene from oil. Moreover, elemental analysis of the biotransformed asphaltene revealed an 80% reduction in the total amount of S and N as compared to control (19). The cell-free supernatant obtained from this biotransformation experiment was also found to biotransform asphaltene and was confirmed to have the presence of extracellular proteins (19). This led us to screen and shortlist enzymes that could play a role in the biotransformation of asphaltene.

Here, we report the use of a bacterial enzyme, thiol peroxidase, to biotransform asphaltene.

The activity of the overexpressed enzyme was assessed and its action on asphaltene was checked using gas chromatography mass spectrometry (GC-MS), Fourier transform infrared spectroscopy (FTIR), and nuclear magnetic resonance (NMR). Changes in the elemental composition, aromaticity, and surface of asphaltene due to enzymatic biotransformation were assessed. This is the first report demonstrating the use of thiol peroxidase in asphaltene biotransformation and porous carbon synthesis.

## RESULTS

### Identification and sequence analysis of thiol peroxidase from *Micrococcus* sp. IITD107

A nine-membered bacterial consortium has been found to biotransform up to 75% of asphaltenes. During this biotransformation, about 80% decrease in the sulfur content of asphaltene was observed (19). Certain genes encoding for enzymes of the peroxidase family, such as heme-containing peroxidase, thiol peroxidase, and dye-decolorizing peroxidase, were found to be present in the genome of the consortium members. Out of the nine bacterial members, five microorganisms, viz. *Lysinibacillus* sp. IITD104, *Sporosarcina* sp. IITD105, *Bacillus* sp. IITD106, *Micrococcus* sp. IITD107, and *Paenibacillus* sp. IITD108, showed the presence of the *tpx* gene encoding the enzyme thiol peroxidase. This was found to be the most abundant peroxidase identified in the consortium (Table S1).

**Fig 1:**
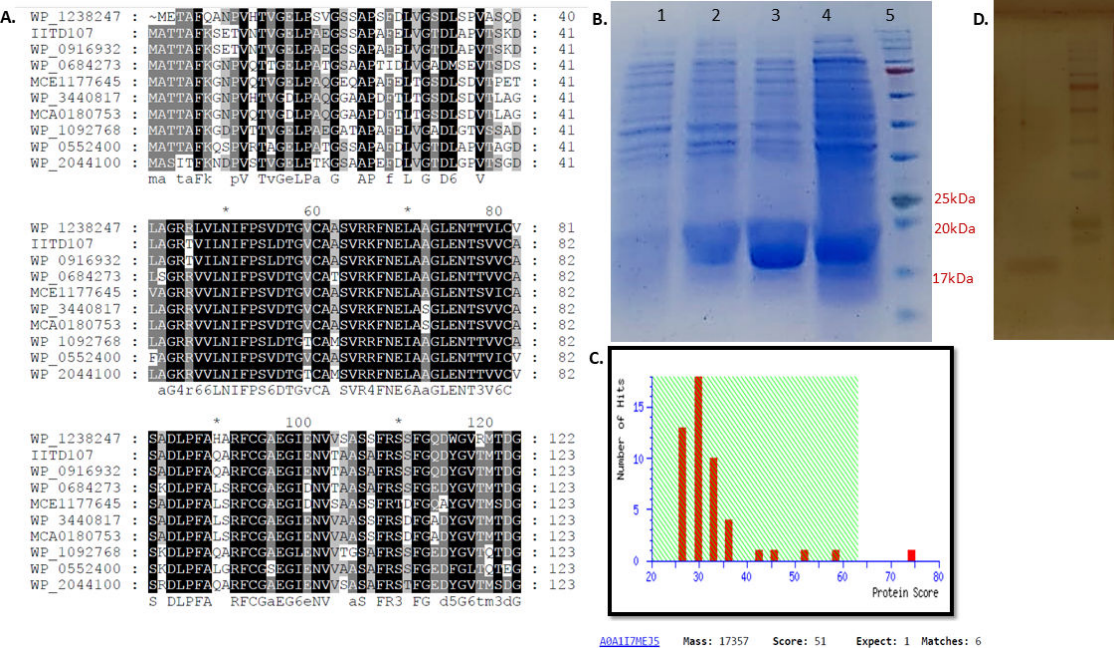
Sequence analysis and SDS-PAGE of protein thiol peroxidase from *Micrococcus* sp. IITD107. (**A**) Multiple sequence alignment using Clustal Omega of protein sequence of thiol peroxidase (our study) from IITD107 (*Micrococcus* sp. IITD107) with WP_1238247 (*Kocuria*), WP_0916932 (*Micrococcus terreus*), WP_0684273 (*Piscicoccus intestinalis*), MCE1177645 (*Micrococcales bacterium*), WP_3440817 (*Nostocoides veronense*), MCA0180753 (*Actinomycetota bacterium*), WP_1092768 (*Brachybacterium endophyticum*), WP_0552400 (*Arthrobacter*), and WP_2044100 (*Brachybacterium muris*). (**B**) SDS PAGE. (lane 1 is crude lysate of uninduced pNP4 in *E. coli*, lane 2 is induced crude lysate of pNP4 in *E. coli* after 3 h of induction with 1 mM IPTG, lane 3 is induced crude lysate of pNP4 in *E. coli* after 5 h of induction with IPTG, lane 4 is induced crude lysate of pNP4 in *E. coli* after 12 h of induction with IPTG, lane 5 is protein marker). (**C**) Mascot score histogram of protein thiol peroxidase. Protein score is −10*Log(*P*), where *P* is the probability that the observed match is a random event. (**D**) SDS-PAGE gel of purified thiol peroxidase enzyme visualized by silver staining.

The nucleotide sequence of the *tpx* gene from *Micrococcus* sp. IITD107, which is of 501 bp in length, was found to show the least percentage identity with nucleotide sequences of the same gene from other members. The maximum identity was found to be about 48% with *Lysinibacillus* sp. IITD104 (Fig. S1). Similarly, the percentage identity matrix of the protein sequence of *Micrococcus* sp. enzyme also showed least similarity with other members of the consortium, with the maximum identity being 47% with *Lysinibacillus* sp. IITD104 (Fig. S2). The enzyme sequence is found to have the thiol peroxidase family signature from the 83rd to the 94th residue as their conserved sites. The sequence analysis of the enzyme for understanding the domain architecture by InterPro revealed the presence of three domains—catalytic, peroxidative, and the dimer interface domain. Three residues—cysteine, arginine, and tyrosine—of the catalytic domain were found to be conserved in the protein. The 60th and 94th residues are cysteines that play a role in the peroxidative and resolving activities of the enzyme (Fig. S3). The multiple sequence alignment of the protein sequence with other thiol peroxidase reported in the literature also shows characteristic conserved domains and conserved cysteine residues of the peroxidase enzyme. The enzyme from *Micrococcus* sp. has a maximum identity of 76% with *Nostocoides veronense* (WP_3440817) and *Actinomycetota bacterium* (MCA0180753) (Fig. 1A).

The gene for thiol peroxidase was amplified from three microorganisms*—Bacillus* sp. IITD106, *Micrococcus* sp. IITD107, and *Paenibacillus* sp. IITD108—and cloned into plasmid pET29a, followed by transformation into the heterologous host *Escherichia coli* Origami (DE3). The expression of the induced protein was checked by SDS-PAGE analysis, and a clear band was observed around the desired molecular weight of 18 kDa (Fig. 1B; Fig. S4 to S6). The overexpressed protein of *Micrococcus* sp. IITD107 was subjected to matrix-assisted laser desorption/ionization time-of-flight (MALDI ToF) analysis, and the protein was confirmed to be thiol peroxidase with a total mass of 17,357 Da. A protein score of 51 out of 80 was obtained. (Fig. 1C)

### Action of various thiol peroxidases on asphaltene

Asphaltene was treated with the crude lysate containing overexpressed thiol peroxidase from the three strains. The amount of asphaltene biotransformation after 18 days of treatment was analyzed, and it was found that the thiol peroxidase from *Bacillus* sp. IITD106 was able to biotransform 48.9% asphaltene, whereas from *Paenibacillus* sp. IITD108, the biotransformation achieved was 45.24%. The maximum asphaltene biotransformation was achieved by the thiol peroxidase from *Micrococcus* sp. IITD107, of about 64.27%, and thus, further studies were carried out using this enzyme (Fig. S7). Molecular docking of asphaltene with the protein thiol peroxidase from two microorganisms, *Micrococcus* sp. IITD107 and *E. coli* (PDB: 3HVS), was performed. Docking score of −196 (confidence score 0.71) and −219 (confidence score 0.8) were obtained, respectively, when docked with a putative archipelago model of asphaltene (Fig. S8A and B). Various hydrophobic and π-stacking interactions were seen between amino acids alanine and phenylalanine and the ligand in the *Micrococcus* sp. protein.

### Biochemical characterization of the recombinant protein

**Fig 2:**
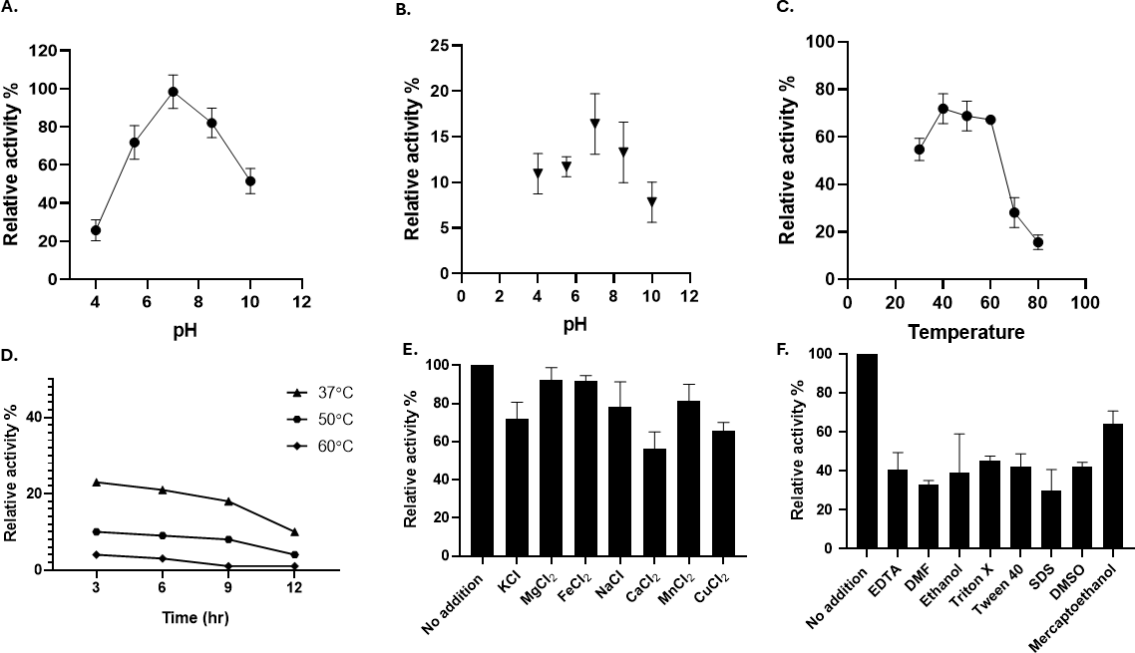
Enzyme kinetics of the recombinant thiol peroxidase. (**A**) Comparative enzyme activity at varying pH of 4.0 to 10.0 was checked as compared to the optimum pH of 7.0. (**B**) Relative activity of enzyme to test pH stability after 24 h of incubation at different pH from 4.0 to 9.0 pH was calculated at pH 7.0. (**C**) The comparative enzyme activity was checked at various temperatures, varying from 20°C to 80°C, as compared to the optimum temperature of 37°C. (**D**) Relative activity of enzyme after incubation at 37°C, 50°C, and 60°C for 3 h, 6 h, 9 h, and 12 h was checked. (**E**) Effect of metal ions on enzyme activity was tested. (**F**) The effect of several detergents and organic solvents on enzyme activity was tested.

The protein was purified and its concentration after purification was 2 mg/mL. The purified protein was analyzed on an SDS-PAGE. A single band of protein was observed on silver staining of the gel (Fig. 1D). The purified recombinant thiol peroxidase was subjected to biochemical characterization with hydrogen peroxide as the substrate. The enzyme activity was found to be 25.5 µM/min·mg with 300 µM of substrate. The *K*_*m*_ value was found to be 28.31 µM along with a *V*_max_ of 3.76 µM/min·mg when plotted on the Hanes-Woolf equation (20, 21). The *K*_*m*_ value was found to be 25.14 µM with a standard error rate of 8.64 along with a *V*_max_ of 510 µM/min·mg and standard error of 28.87, when plotted on the Michaelis-Menten equation, along with a *K*_cat_ value of 152.94 s^−1^. The Michaelis-Menten plot is shown in Fig. S9. The 95% confidence interval obtained was 460 to 565.7 for *V*_max_, whereas for *K*_*m*_, the range obtained was 13.2 to 42.55 along with a standard error of 8.64. The pH and temperature profiles were further analyzed using the same substrate. The enzyme exhibited maximum activity at pH 7.0, which was considered to be 100%. The enzyme was also functionally active in the pH range of 6.0 to 9.0, and its relative activity varied from about 70% to 80% as compared to its maximum (Fig. 2A). The pH stability of the enzyme was checked at pH 7.0 after 24 h of incubation in different buffers, and the enzyme was found to exhibit only 16.4% relative activity at pH 7.0 and 13.2% relative activity at pH 8.5 (Fig. 2B). The enzyme thiol peroxidase was found to be active over the temperature range of 30°C to 60°C with maximum activity at about 40°C (Fig. 2C). The enzyme was found to have stability at 37°C for about 6 h, after which the activity was reduced, whereas the enzyme had very little stability at 50°C and 60°C (Fig. 2D). The addition of organic solvents and detergents was found to reduce the activity of the enzyme in this study, whereas the metal ions, too, were found to have no additional activator effect on the enzyme. The divalent metal ions Mg^2+^, Fe^2+^, and Mn^2+^ had minor effects on the activity of the enzyme and were almost at par with values of 92.18%, 91.68%, and 81.25%, respectively, whereas the metal ions of K^+^, Na^+^, Ca^2+^, and Cu^2+^ reduced the activity to 71.87%, 78.12%, 56.25%, and 65.2%, respectively, of its maximum potential. This indicated the metal ion-independent nature of the enzyme and also its resistance to heavy metal load (Fig. 2E). The organic reagents EDTA, dimethylformamide (DMF), ethanol, dimethyl sulfoxide (DMSO), and ß-mercaptoethanol were found to reduce the enzyme activity to 40.62%, 32.81%, 39%, 42.18%, and 64.06%, whereas the detergents Triton X, Tween 40, and SDS also reduced the activity to 45.31%, 42.18%, and 29.68%, respectively (Fig. 2F). However, no loss of activity was seen with any of the reagents, and it suggests that the enzyme is able to withstand environmental stress. To enhance the stability of purified enzyme, polyethylene glycol was added, and it was found that the enzyme was stable until 18 h, with a relative enzyme activity of 45% (Fig. S10).

### Thiol peroxidase altered the components in asphaltene

**Fig 3:**
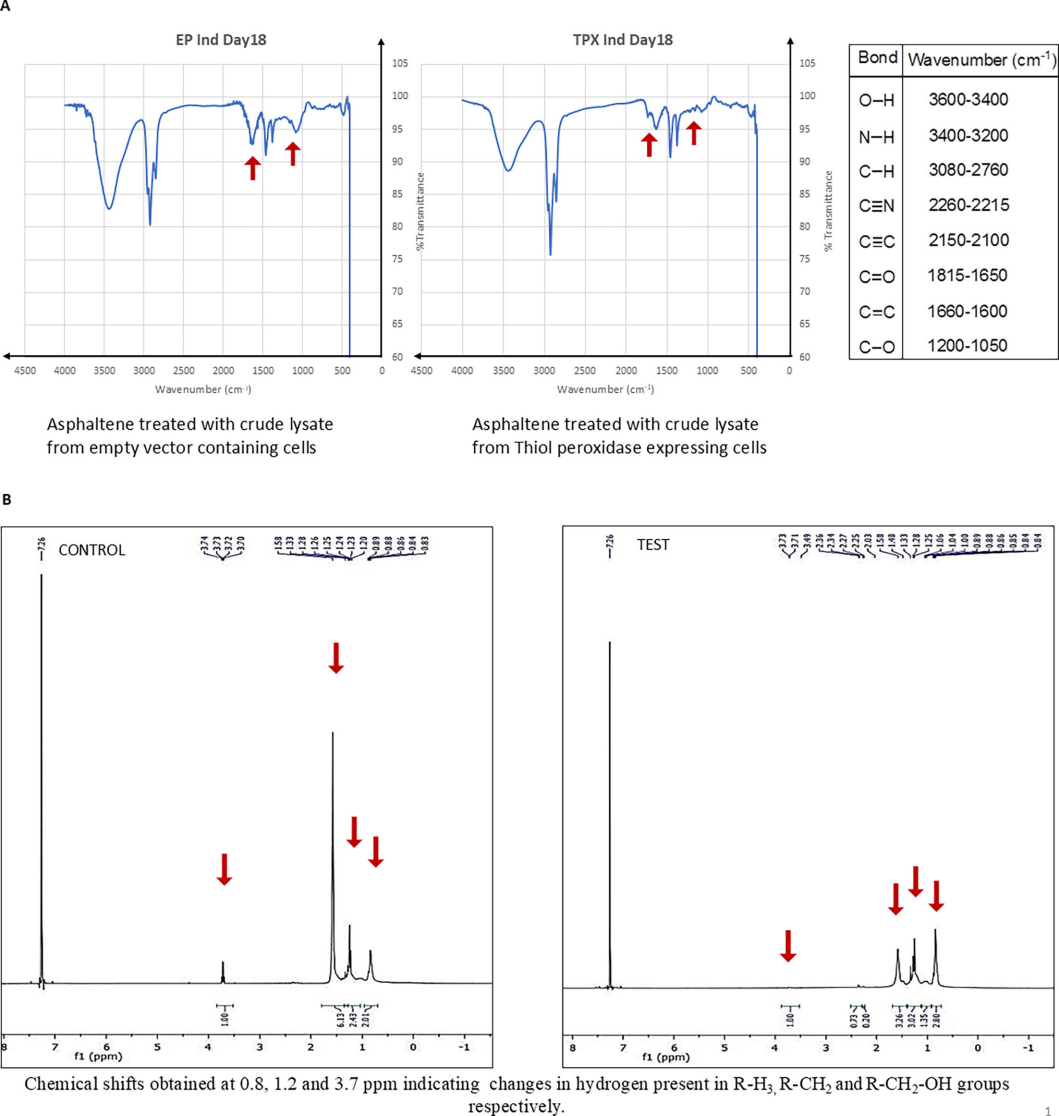
Changes in asphaltene observed. (**A**) FTIR spectra of asphaltene fraction, untreated with enzyme as compared to treated with overexpressed enzyme. (**B**) NMR spectra of control sample (asphaltene untreated with enzyme) as compared to test sample (asphaltene treated with overexpressed enzyme).

Asphaltene was treated with the crude lysate containing overexpressed enzyme and its biotransformation was monitored over a period of 18 days. The FTIR analysis of the day 18 sample and control was carried out. Compared with the control, there were prominent changes in the FTIR spectra of the test samples. The FTIR analysis revealed incorporation of oxygen corresponding to bond C = O at wavenumber 1,734 cm^−1^, which was seen in test sample but absent in control (Fig. 3A). Peaks corresponding to the C-O bond were also found to be altered in the test sample. This indicates changes in the bonds present in the target due to the action of the enzyme. The H^1^ NMR spectra revealed chemical shifts obtained at 0.8, 1.2, and 3.7 ppm, showing changes in hydrogen present in R-H_3_, R-CH_2_, and R-CH_2_-OH groups, respectively (Fig. 3B). This further confirms the action of the peroxidase enzyme on asphaltene molecules.

### Analysis of the enzymatic biotransformation products

**Fig 4:**
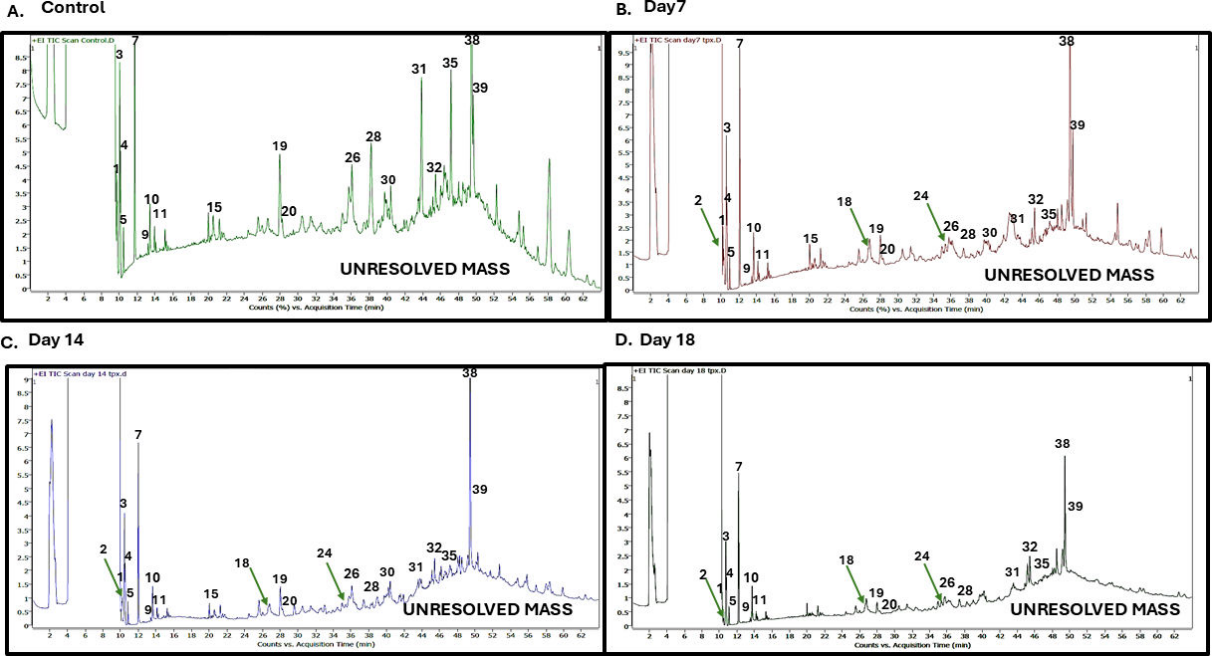
GC-MS chromatograms of asphaltene indicating a decrease in the size of peaks corresponding to several metabolites over a period of time (**A**). Control asphaltene after 18 days, untreated with enzyme. (**B**) Asphaltene fraction after 7 days of treatment with overexpressed enzyme. (**C**) Asphaltene fraction after 14 days of treatment with overexpressed enzyme. (**D**) Asphaltene fraction after 18 days of treatment with overexpressed enzyme. A few of the prominent peaks reducing in size over incubation time have been labeled. A detailed list of the decreasing peaks is mentioned in Table S2, and the labeling has been done accordingly in the chromatograms. Green arrows depict the presence of new peaks at days 7, 14, and 18, which were absent in control samples.

To analyze the degradation products, GC-MS was performed on asphaltene samples treated with crude cell lysate containing the overexpressed enzyme collected over a duration of 18 days. It was observed that there was a clear reduction in the size of the various peaks over the period of incubation obtained in the chromatogram (Fig. 4). The untreated samples of asphaltene had the presence of unresolved mass (Fig. S11). This suggests that the overexpressed enzyme indeed had an effect on the components of asphaltene.

Compared with the GC-MS spectra of the control asphaltene, which consisted of untreated and raw asphaltene at day 0 (Fig. S11A), asphaltene treated with crude lysate of only wild-type *E. coli* cells at day 18 (Fig. S11B), and asphaltene treated with crude lysate of *E. coli* cells containing an empty plasmid vector at day 18 (Fig. 4A), the various test samples showed the presence of compounds such as benzene, 1,3-dimethyl (5), octadecane, 6-methyl- (12), decane, 2,4,6-trimethyl- (13), dodecane, 2,6,10-trimethyl- (16), benzothiazole, 2-(2-hydroxyethylthio)- (24), and octadecanoic acid, 2-propenyl ester (40), along with several others. Several comparatively smaller-sized compounds, such as benzene, [3-(2-cyclohexylethyl)-6-cyclopentylhexyl]- (2), ethylbenzene (1), *p*-xylene (4), benzaldehyde (10), benzyl alcohol (9), 2,4-difluorobenzene, 1-benzyloxy- (11), benzeneacetic acid, 2-tridecyl ester (14), nonadecane (23), *n*-hexadecanoic acid (25), phenol, 4,4´-(1-methylethylidene)bis- (30), 13-docosenamide, (Z)- (38), hexadecane (20), tetradecane, 2,6,10-trimethyl- (26), and tetracontane (35), were found to be reduced at all time points compared with the control, along with several others (Fig. 4B through D). Reduction in the size of several peaks and their area corresponding to heptadecane, 2,6,10,14-tetramethyl- (22), 13-docosenamide, (Z)- (38), and octadecane, 3-ethyl-5-(2-ethylbutyl)- (39) was seen after 7 days of treatment. Interestingly, three new peaks were observed, representing the compounds benzene, [3-(2-cyclohexylethyl)-6-cyclopentylhexyl]- (2), 3-bromo-4-methyl-2,3-dihydrothiophene, 1,1-dioxide (18), and benzothiazole, 2-(2-hydroxyethylthio)- (24). The peaks for benzenepropanoic acid, α-(hydroxyamino)- (15), 2-methoxymyristic acid (29), phenol, 4,4´-(1-methylethylidene)bis- (30), and octadecanoic acid, 2-propenyl ester (40) were present until day 14 but absent on day 18 (Table S2).

### Decrease in sulfur and nitrogen content was observed after asphaltene biotransformation

**Fig 5:**
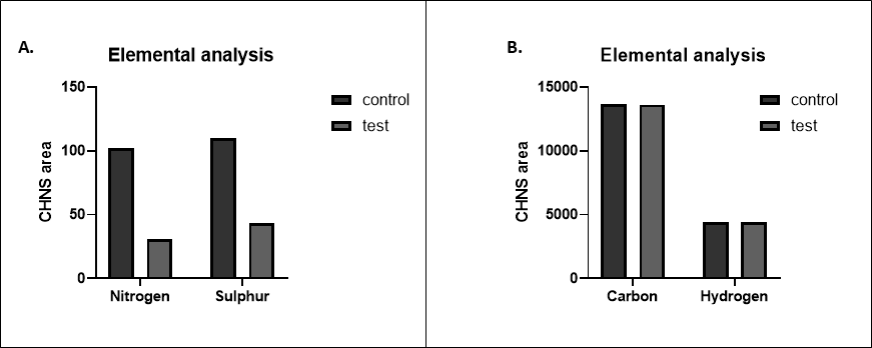
Elemental analyses of treated asphaltene fraction as compared to control, analyzing the changes in elemental composition of carbon, hydrogen, nitrogen, and sulfur. (**A**) Comparative elemental analysis with respect to sulfur and nitrogen, (**B**) with respect to carbon and hydrogen.

The elemental composition of the enzyme-treated asphaltene sample, compared to that of untreated asphaltene sample, depicted a reduction in about 69% nitrogen and 60% sulfur content (Fig. 5A). No significant changes were obtained in the carbon and hydrogen content of the test samples as compared to control (Fig. 5B). This is indicative of the maintenance of the calorific value of the substrate. The enzyme acts on the nitrogen and sulfur moieties, which are present as heteroatoms in the complex structure of asphaltene.

### Decrease in the aromaticity of asphaltene

**Fig 6:**
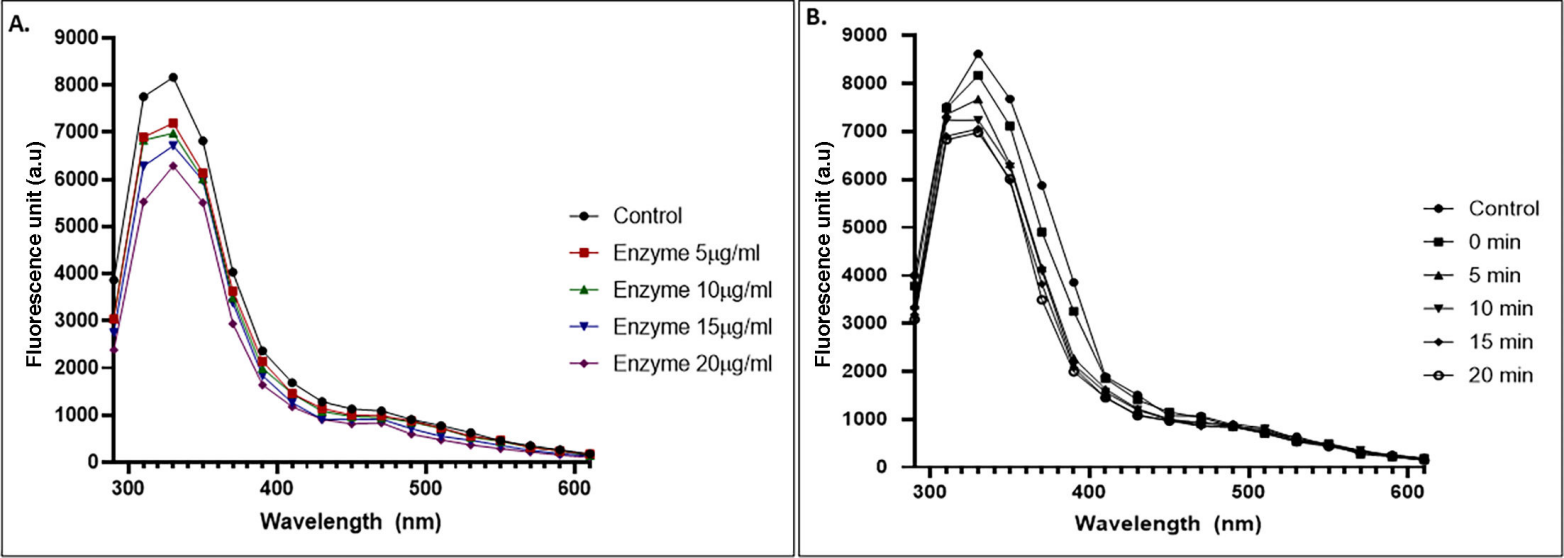
Fluorescence emission spectra obtained from asphaltene. (**A**) As a result of increasing concentration of enzyme, the fluorescence emission obtained was found to reduce. (**B**) A decrease in the fluorescence emission was observed with increasing incubation times of enzyme with asphaltene.

**Fig 7:**
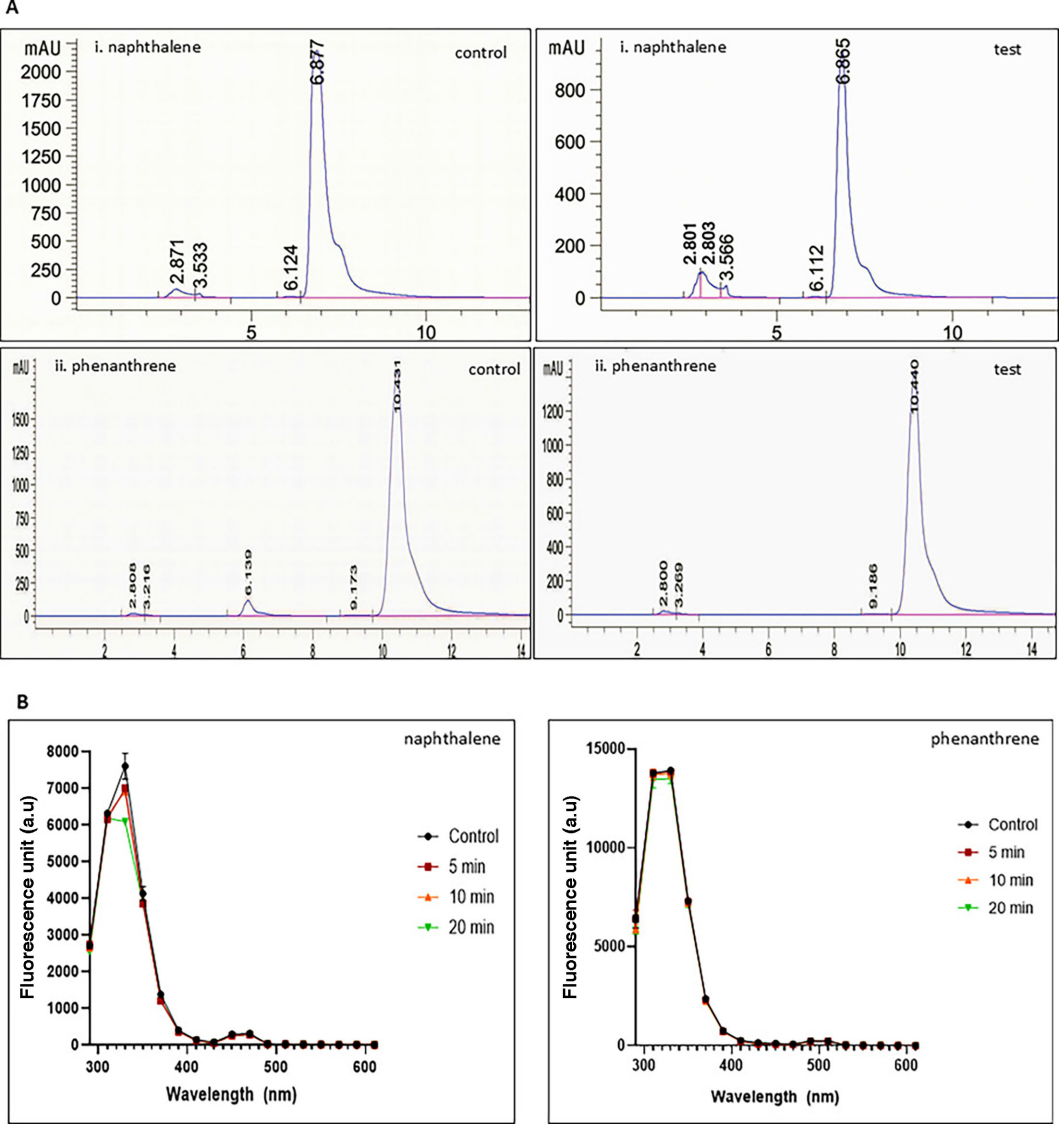
(**A**) HPLC chromatograms of untreated and treated PAH compounds. (i) Naphthalene shows a 67.8% decrease in its concentration. (ii) Phenanthrene shows a 23.5% decrease in concentration. (**B**) Aromaticity decrease observed in pure PAHs naphthalene and phenanthrene after treatment with thiol peroxidase.

As the structure of asphaltene has the presence of several polyaromatic rings, due to biotransformation, it is likely that a reduction in aromaticity would occur. Purified enzyme was added to pure asphaltene, and the decrease in aromaticity levels as compared to untreated control samples was tested. It was found that with increasing incubation period and increasing concentration of enzyme, a visible reduction in the asphaltene aromaticity level was observed (Fig. 6). The action of purified enzyme was also checked on lignin, but no change in lignin was observed even after 24 h of treatment (Fig. S12). Thus, to check the effect of enzyme on aromaticity, pure PAHs, such as naphthalene and phenanthrene, were used. The concentration of naphthalene and phenanthrene was found to be reduced by 67.8% and 23.5%, respectively by high performance liquid chromatography (HPLC), as compared to control when treated with pure enzyme (Fig. 7A). The aromaticity of the compounds was also found to reduce as a result of treatment with enzyme (Fig. 7B). The turnover number of this enzyme with respect to asphaltene has been found to be 13.2 s^−1^.

### Biotransformation of asphaltene in a packed bed column

To scale up and develop a continuous process, we prepared a packed bed column, which was packed with purified and immobilized thiol peroxidase enzyme. About 40 mL model oil containing 300 mg asphaltene was passed through it. The treated fraction was collected and weighed, and a reduction of 34% weight was observed in 6 h as compared to the untreated fraction (Table S3). The biotransformation efficiency was calculated by measuring absorbance of residual asphaltene at 300 nm. It was determined that about 30% biotransformation occurred in the treated fraction due to the action of enzyme as compared to that of the untreated fraction (Fig. S13). The action of the enzyme on 20 mL crude oil was also checked, and it was found that the total oil was collected in 48 h and about 28% asphaltene was biotransformed with a 23.5% reduction in weight (Fig. S13; Table S3). The stability of the enzyme after immobilization was checked, and it was found to have a relative activity of 32% after 24 h (Fig. S14A).

### Asphaltene treatment with thiol peroxidase resulted in formation of porous carbons

**Fig 8:**
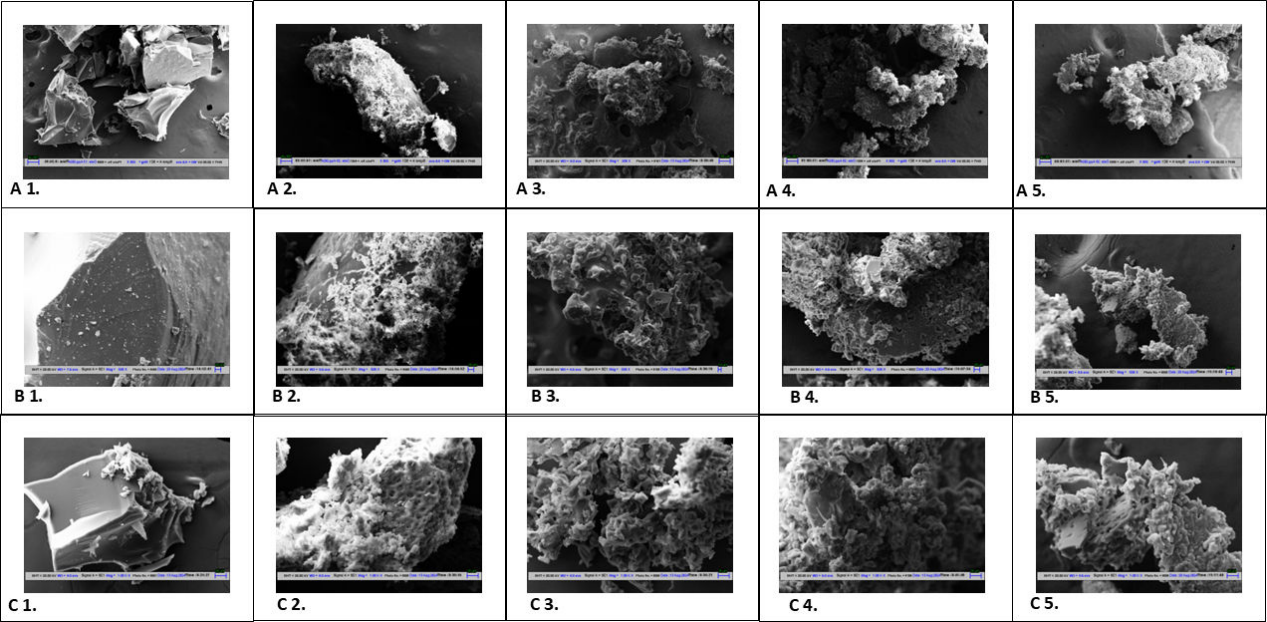
SEM images of untreated and treated asphaltene fraction. (**A**) Imaging at magnification of 200×. (**B**) Imaging at magnification of 500×. (**C**) Imaging at magnification of 1,000×. (1) Pure and untreated asphaltene as control sample. (2) Imaging at day 3 of treatment with overexpressed enzyme. (3) Imaging of asphaltene treated for 7 days with enzyme. (4) Imaging of treated asphaltene after 14 days of incubation with enzyme. (5) Imaging after 18 days of treatment with overexpressed thiol peroxidase enzyme.

In order to study the physical changes on asphaltene, scanning electron microscopy (SEM) of thiol peroxidase-treated samples was performed with respect to control. The control samples of raw asphaltene showed a smooth surface appearance at all magnifications (Fig. 8A1, B1 and C1). At 200× and 500× magnifications, the presence of both untransformed and transformed regions of asphaltene was observed until the 14th day of incubation (Fig. 8A2, B2, A3, and B3). The samples after day 3 appeared porous (Fig. 8C2), day 7 showed the disruption of pores (Fig. 8C3), and day 14 showed a further agglomerated-like appearance (Fig. 8C4). The presence of porous structures along with disrupted and fragmented asphaltene moieties has been observed until the 18th day of treatment (Fig. 8C5). The results suggest that asphaltene biotransformation by thiol peroxidase affected the smoothness of the surface. The asphaltene surface appeared rough and agglomerated after treatment with thiol peroxidase. Interestingly, the treatment of asphaltene using enzyme revealed the formation of large pores in the target substrate. Thus, thiol peroxidase can also be used for the synthesis of porous carbons from asphaltene, which in turn finds numerous applications in bioremediation.

## DISCUSSION

In the current study, the enzyme thiol peroxidase has been studied for the first time to have an effect on the complex polyaromatic petroleum hydrocarbon—asphaltene. Asphaltenes have been a major problem of the petroleum industry, and several physical and chemical methods for their removal have been attempted for many years (22, 23). Biological methods of asphaltene removal provide an eco-friendly way of upgrading heavy oil. Few bacteria and fungi have been reported to act on asphaltene, but the time required for degradation of this recalcitrant compound was longer (1). A nine-membered bacterial consortium has been reported recently that is able to biotransform about 75% of asphaltene (19). The consortium members have the genes for certain peroxidases, out of which the gene for thiol peroxidase was the most abundantly present, in a total of five out of the nine bacterial members.

Peroxidases are considered to be part of the initiating microbial degradation steps along with other redox enzymes such as mono- and dioxygenases (24). Lignin peroxidase, manganese peroxidase, and dye-decolorizing peroxidase are reported to have biodegradation capabilities (25). Thiol peroxidases are abundantly present in several organisms varying from bacteria, fungi, and plants such as *Pseudomonas aeruginosa* (26), *Mycobacterium tuberculosis* (27), *Helicobacter pylori* (28), *Saccharomyces cerevisiae* (29), and *Arabidopsis thaliana* (30) but are not reported for polyaromatic hydrocarbon degradation potential yet. The peroxiredoxin enzyme is involved in peroxide scavenging and hence present in many organisms. The enzyme acts in intracellular oxidative stress defense as well as in cellular signaling (31, 32). Thiol peroxidase belongs to the peroxiredoxin family of enzymes which makes use of an electron donor—thioredoxin (Trx), in its reduced form—for catalytic reduction of the substrate (33). Thiol peroxidase belongs to the non-heme-dependent peroxidase family along with other members such as halo peroxidase, NADH peroxidase, manganese catalase, and alkyl hydroperoxidase (34). Out of the total peroxidases identified in nature, heme-free peroxidases are about 20% in abundance (35). Thiol-specific antioxidant proteins have been reported in yeast, human erythrocyte, and rat brain, which are capable of oxidizing H_2_O_2_ and have two cysteine residues conserved (36).

The peroxidases that utilize thioredoxin as a source of electron for reduction of hydrogen peroxide along with oxidation of thiols belong to the glutathione class of peroxidases (37, 38). The oxidized resultant thioredoxin is cycled back to the reduced form by the thioredoxin reductase reaction which utilizes reductant NADPH (26, 39). The detailed mechanism of reaction is shown in Fig. S14B. The proposed mechanism of action of enzyme with asphaltene is shown in Fig. S15. Bacteria reported for growth on xenobiotics such as polychlorinated biphenyls result in generation of oxidative stress (40). Such a stress serves as a stimulus for thiol peroxidases. The H_2_O_2_ and reactive oxygen species (ROS) are the toxic byproducts of oxygen-consuming enzymes such as mono- and dioxygenases responsible for the aerobic degradation of aliphatic and aromatic hydrocarbons. It is likely that the thiol peroxidases are induced in response to the oxidative stress in the bacterium due to the exposure to aliphatic or aromatic hydrocarbons. In a study by Dosanjh in *Mycobacterium tuberculosis*, higher expression of thiol peroxidase was observed when subjected to thiol stress. Redox cyclic agents, superoxides, and diamides have been found to play the role of stronger inducers of *tpx* in the strain as compared to its peroxide substrates like hydroperoxides (41).

The 18 kDa thiol peroxidase from the *Micrococcus* sp. IITD107 belongs to the thioredoxin class of enzyme with two conserved cysteine residues. One cysteine residue reacts with the peroxide and forms a sulfenic acid intermediate, while the other cysteine acts on this acid and ultimately forms a disulfide bond. The disulfide is then reduced to a dithiol using an NADPH molecule (42).

The sequence analysis of other members of the peroxiredoxin family also shows the conserved domains of the thiol peroxidase family. Purification of the recombinant protein enabled us to check its activity. The enzyme was found to efficiently reduce hydrogen peroxide. From the literature, depending on the oxidation of NADPH, the Dalziel equation reported the thiol peroxidase from *Mycobacterium tuberculosis* to have a *K*_cat_ value of 11.1 s^−1^ (43), while the one from *Pseudomonas aeruginosa* has a *K*_cat_ value of 26.6 s^−1^ (26), as indicated from its Michaelis-Menten plot. The kinetic study of a thiol peroxidase from *E. coli* gave a *K*_cat_ value of 76.0 s^−1^ determined by the Hanes-Woolf equation. The *K*_cat_ value of 152.94 s^−1^ of thiol peroxidase determined in our study was higher than those reported. Thus, the higher *K*_cat_ value indicates high efficiency of the enzyme. The Hanes-Woolf equation is used in case of a bisubstrate ping-pong reaction mechanism of the enzyme (20). The peroxiredoxin family members have been known to fit the best to a ping-pong mechanism, and hence this is the preferred method for determining enzyme kinetics (21). The *K*_*m*_ value of *E. coli* thiol peroxidase was determined to be 1,730 µM with H_2_O_2_ and about 25.5 µM with Trx1 as compared to the *K*_*m*_ value of 28.31 µM of the enzyme in this study, indicating high affinity toward the substrate (20).

The enzyme in our study was found to be active over a broad temperature and pH range of 30°C to 60°C and pH of 6.0 to 9.0. The enzyme thus shows activity in acidic, neutral, as well as alkaline conditions, which can make it useful for a wide range of applications under different environmental conditions. The enzyme is comparable to the thermostable plant peroxidase from the leaf of *Citrus medica*, showing a maximum activity with guaiacol at a pH of 6.0 and 50°C, and is active over a range of acidic as well as basic pH from pH 4.0 to 8.0, along with a temperature range of 30°C to 80°C (44). The NADPH peroxidase from a sulfate-reducing bacteria was reported to be most active over the pH range of 6.5 to 7.0 and with the optimum temperature of 37°C. The enzyme had a bell-shaped curve as a function of both temperature and pH (45), which is similar to that of the enzyme of our study.

Unlike other peroxidases such as that from *Actinomyces viscosus*, which was found to have a positive effect on the enzyme activity when 10 mM of Fe^2+^, Ca^2+^, and Mn^2+^ was added and a negative effect on its activity due to Mg^2+^, Hg^2+^, and Cu^2+^ (46), the enzyme in our study was found to be majorly unaffected by the addition of metal ions. As crude oil has the presence of several metal ions, the effect of various metal ions on enzyme activity was checked. The ions of iron, calcium, sodium, copper, manganese, and magnesium are present in crude oil in varying amounts (47–49). The activity was found to be reduced in case of solvents such as ethanol, reducing agents such as DMF and ß-mercaptoethanol, and oxidants such as DMSO. The chelating agent EDTA as well as detergents such as Tween 40, Triton X, and SDS also reduced the activity of enzyme. This indicates that the enzyme is able to function on its own without the requirement of detergents.

The enzyme was able to act on the asphaltene, and a reduction in the number of peaks obtained in the control samples was seen over a period of 18 days in the GC-MS spectra. The maximum biotransformation of asphaltene was achieved by the enzyme produced by the *Micrococcus* sp. IITD107. The thiol peroxidase from *Bacillus* sp. IITD106 and *Paenibacillus* sp. IITD108 was also able to cause biotransformation up to some extent. The *in silico* docking analyses of thiol peroxidase from *Micrococcus* sp. IITD107 and *E. coli* revealed comparable docking scores of −196 and −219. The PLIP server indicated hydrophobic interactions and π stacking between the amino acids from the *Micrococcus* protein and ligand, whereas hydrophobic interactions and π cation interactions were seen in amino acids from the *E. coli* protein and the ligand. On analysis of the effect of biotransformation from the thiol peroxidase from *Micrococcus* sp. IITD107, it was found that the area of unresolved mass seen in the chromatogram was largely reduced by the end of 18 days of incubation with the enzyme as compared to controls (Fig. S11). A study involving biotreatment of asphaltene using microorganism on several known biomarker compounds has also revealed a reduction in the number of peaks obtained in GC-MS spectra post-biotreatment (50). Several small-sized aromatic hydrocarbons such as ethylbenzene, benzyl alcohol, benzaldehyde, and xylene were observed by the end of the 18th day of incubation with the overexpressed enzyme. Similarly, linear aliphatic chain compounds such as nonadecane and pentadecane have been observed. Previously, n-alkanes such as dodecane, tetradecane, and hexadecane, along with several others, have been obtained as degradation products from oily sludge containing asphaltene (51). Compounds such as octadecanoic acid and hexadecanoic acid have also been obtained, indicating further transformation of the linear aliphatic chains (52). A representative structure of asphaltene, which has been proposed, considers the complex structure to be made of nine subcomponents, likely to be dibenzotetracene, thiooxane, methyl–napthochrysene, (tetrabutyl, phenyl, methylene) dibenzene dibenzochrysene, dibenzothiophene, carbazole, anthracenedione, and methyl nitrophenyl fluorene (19). Also, the island and archipelago model of asphaltene structure suggests the compound to be made up of a group of polyaromatic ring structures which are connected by aliphatic chains (53). The obtained small-sized aromatic ring and alkane chain degradation products, which are detected throughout the course of enzymatic treatment of asphaltene from this study, could be a result of the breakdown of the complex polyaromatic hydrocarbons present in the parent structure.

On analyzing the extracted asphaltene samples, changes in the functional groups as well as chemical shifts in peaks corresponding to several bonds were observed in the FTIR and NMR spectra. Similar studies related to biodegradation of asphaltene by bacterial cultures have revealed changes in the FTIR spectra of asphaltene (50, 51). The elemental analysis also confirmed a reduction in the total amount of nitrogen and sulfur present in the test samples post-treatment with enzyme as compared to the control samples while maintaining the carbon and hydrogen content. As calorific value describes the combustion value of fuel (54), the maintenance of calorific value is desirable. The change in elemental composition suggests the targeted action of the thiol peroxidase enzyme on nitrogen and sulfur residues present in asphaltene. Due to the large number of aromatic structures present in asphaltene, it has a very high aromaticity level. The aromaticity was found to reduce as a result of the addition of pure enzyme to asphaltene. A chloroperoxidase was also found to reduce the aromaticity of asphaltene (55). The maximum emission was obtained at 330 nm, and the fluorescence intensity decreased with the increasing amount of enzyme and increasing incubation time. This indicates that the biotransformation of asphaltene leads to a change in the number of polycyclic aromatic structures present in the complex molecule of asphaltene, and a reduction in the polycyclic structures indicates a reduction in aromaticity. The several small-sized polyaromatic hydrocarbon and alkane chain compounds obtained post-treatment of asphaltene with the enzyme are indicative of a successful biotransformation of the complex hydrocarbon compound. Lignin is known to have a very complex structure consisting of several polyaromatic ring structures, and hence the action of thiol peroxidase on lignin was tested. It was seen that even after 24 h of treatment, no biotransformation occurred. The reason behind the enzyme’s inability to act on alkali lignin is not clear at present. Attempts have been made to modify alkali lignin to enhance enzyme accessibility with the substrate for increased enzymatic reaction (56). However, the enzyme was able to reduce aromaticity levels of smaller PAH compounds such as naphthalene and phenanthrene. These compounds have the presence of two and three aromatic rings in their structure. An engineered fungal peroxygenase has been previously reported to act on naphthalene to reduce its aromaticity (57). A peroxidase from fungus *Agrocybe aegerita* was able to form 1-naphthol and 1,4-naphthoquinone by reacting with naphthalene, suggesting its capability of hydroxylating aromatic rings. Such incorporation of oxygen in the aromatic ring is often referred to as the phenomenon of “peroxidase shunt,” in which the origin of oxygen is H_2_O_2_ (58). Manganese peroxidase and laccase have been previously reported for phenanthrene oxidation (59).

The reduction in aromaticity due to the action of thiol peroxidase further suggests the action of this enzyme on aromatic ring compounds. Certain enzymes have been found to produce a protein radical as an intermediate during their enzymatic reactions (60). This protein radical forms a crosslink between the protein’s amino acid residue and the target substrate. Further oxidation of the product occurred in the presence of H_2_O (61). It is likely that the enzyme thiol peroxidase also forms a protein radical in the presence of peroxide and carries out the oxidation of target aromatic substrate. The protein radical might involve the cysteine residue to form a crosslink with the substrate. Peroxidases such as lignin peroxidase and manganese peroxidase have been well established to degrade polyaromatic hydrocarbons (62, 63); however, this is the first report on the action of thiol peroxidase for asphaltene biotransformation.

Interestingly, it was observed that the surface of asphaltene became completely porous after 3 days of treatment with thiol peroxidase (Fig. S16). They can serve as porous carbons for various applications. The materials of porous carbon have been applied to the fields of adsorption, catalysis, carbon capture, as well as energy storage due to several properties such as high surface area, mechanical strength, high conductivity, chemical stability, thermal conductivity, and low density. The tunable properties of porous carbon, such as pore size and variable functional groups, are the major reasons for their wide applications (64, 65). The recalcitrant fraction asphaltene is a rich biomass of carbon. Utilization of the enzyme thiol peroxidase on this substrate can lead to the formation of pores, which in turn can make asphaltene a suitable fraction for development of porous carbon material (65). To date, synthesis of porous carbons has been reported by chemical methods (66). This is the first report on the biological synthesis of porous carbons using thiol peroxidase. Thus, this study further opens up the potential application of this enzyme—thiol peroxidase—not only for the biotransformation of several other polyaromatic hydrocarbons, but also for the synthesis of porous carbons. The use of a packed bed reactor with immobilized enzyme thiol peroxidase can be used in the refinery settings to treat asphaltene and carry out its valorization into porous carbon.

## MATERIALS AND METHODS

### Growth conditions and media used

The strains of *E. coli* (DH5α and Origami DE3) were cultured at 37°C at 180 rpm in Luria-Bertani (LB) broth (Himedia, India). The concentration of the antibiotic kanamycin (Himedia, India) used in the media was 50 µg/mL. The *Micrococcus* sp. IITD107 (MTCC 25278) was cultured in LB broth at 30°C and 180 rpm.

### Cloning of thiol peroxidase gene

The plasmid pET29a was isolated and digested with the restriction enzymes NdeI and HindIII. The genomic DNA of *Bacillus* sp. IITD106, *Micrococcus* sp. IITD107, and *Paenibacillus* sp. IITD108 was manually isolated using the phenol-chloroform method of DNA extraction (67). The gene *tpx* coding for the enzyme thiol peroxidase was amplified from the genome using gene-specific primers (Table S4). The amplified gene product was purified and incubated with NdeI and HindIII overnight for digestion. The plasmid vector and the digested insert were ligated, and the ligation mixture was transformed into *E. coli* DH5α to obtain Kan^R^ colonies. The colonies obtained were subjected to colony PCR using the insert-specific primers, and plasmid was isolated from the positive colonies. The isolated plasmid was subjected to restriction digestion to check the release of the desired gene product and confirmed by Sanger sequencing.

### Expression of protein

To study the expression of the cloned enzyme, the plasmids pNP4, pNP7, and pNP8 were transformed into the *E. coli* expression host Origami DE3. The culture was then induced with 1 mM isopropyl β-D-1-thiogalactopyranoside (IPTG). Cell pellets were collected in microcentrifuge tubes after 3 h, 5 h, and overnight incubation following IPTG induction. The cell pellet was mixed with 80 µL SDS loading dye and heated at 95°C for 10 min. The tubes were then centrifuged at 12,000 rpm for 10 min. The supernatant obtained was loaded onto SDS-PAGE gel and run at 100 V for 2.5 h. The gel was stained with Coomassie brilliant blue stain for 1 h followed by destaining for 30 min.

### MALDI TOF

The protein from the SDS-PAGE gel was sliced to small pieces and was subjected to complete destaining and drying. It was then rehydrated using dithiothreitol (DTT) followed by incubation steps with iodoacetamide and ammonium bicarbonate and again dehydrated. It was subjected to overnight incubation at 37°C with trypsin solution. The gel piece was extracted thrice with an extraction buffer containing acetonitrile and trifluoroacetic acid followed by complete drying. The dried premix was resuspended in buffer containing acetonitrile and trifluoroacetic acid, and the peptides obtained were mixed with HCCA (α-cyano-4-hydroxycinnamic acid) matrix. About 2 µL of the sample was loaded onto a MALDI plate. It was analyzed in the MALDI/TOF Bruker Daltonics ULTRAFLEX III instrument. The software Flex Analysis was used to obtain the peptide mass fingerprint. The mass obtained was submitted to the Mascot search engine for identification of the protein.

### Purification of protein and visualization

The plasmid pNP4 was transformed into the *E. coli* Origami strain. The culture was induced with IPTG at an OD_600_ of 0.6. The cell pellet was obtained by centrifuging the cells at 5 h from induction at 7,000 rpm for 10 min. Pure protein was obtained by the process of Ni-NTA purification followed by gel filtration. The cell lysate was suspended in binding buffer containing 20 mM sodium phosphate, 0.5 M NaCl, and 5 mM imidazole, pH 7.4. HisTrap HP column was used, and the volume was 5 mL. The flow rate was maintained at 1 mL/min. About two column volumes of binding buffer were passed followed by washing with five column volumes of wash buffer (20 mM sodium phosphate, 0.5M NaCl, 5 mM imidazole, pH 7.4). About two column volumes of elution buffer were used for elution which contained 20 mM sodium phosphate, 0.5 M NaCl, and 0.5M imidazole at pH 7.4. Post-elution, 500 µL of sample volume in eluent containing about 25 mg/mL protein was filtered through a 0.22 µm filter and centrifuged at 10,000 *g* for 10 min. The sample was then loaded in a column equilibrated with one column volume equilibration buffer containing 20 mM MES and 150 mM NaCl. Superdex 200 (10/300) column was run for five column volumes of buffer, and all fractions were collected with a flow rate of 0.75 mL/min. The protein was visualized by silver staining.

### Enzyme assay and kinetics

The peroxidase activity was tested using the NADPH-linked peroxidase activity assay (68). Pure Trx and thioredoxin reductase (TrxR) from *E. coli* were obtained from Sigma-Aldrich, and the peroxidase activity linked to oxidation of NADPH via the Trx-TrxR system was monitored by recording a decrease in absorbance at 340 nm using an Eppendorf Biospectrophotometer. The reaction mixture had 50 mM HEPES [4-(2-hydroxyethyl)-1-piperazine ethanesulfonic acid]-NaOH buffer (pH 7.0), 20 µg Trx, 6.25 µg TrxR, 20 µM NADPH, and 10 µg purified protein with varying concentrations of hydrogen peroxide. The total volume of reaction was 1 mL, and the enzyme assay was carried out at room temperature. For each substrate concentration, the data obtained were plotted according to the Michaelis-Menten equation in order to get the *V*_max_, *K*_*m*_, and *K*_cat_ values on GraphPad Prism v.8.0.2. For each substrate concentration, the data obtained were also plotted according to the Hanes-Woolf representation of the Michaelis-Menten equation in order to get the *V*_max_, *K*_*m*_, and *K*_cat_ values (20, 21).

For optimal pH determination, buffers of varying pH were used. For pH 4.0, a 50 mM acetate/NaOH buffer was used; for pH 5.5, 50 mM MES (2-(N-morpholino)ethanesulfonic acid sodium salt)/NaOH buffer was used; and for pH 7.0, 50 mM HEPES-NaOH buffer was used. The buffer Tris-HCl (50 mM) was used for pH 8.5, and 50 mM glycine/NaOH was used for pH 10. For determining the pH stability, the enzyme was incubated at varying pH for 24 h at 4°C before checking for the activity at pH 7.0 in the reaction mixture stated above. The enzyme was incubated in different buffers of varying pH and was reconstituted in HEPES-NaOH buffer of pH 7.0 after carrying out conventional protein precipitation using 20% trichloroacetic acid-acetone to the protein sample in a ratio of 1:4. The effect of various temperatures on enzyme activity was checked by incubating the buffer at varying temperatures before checking for the relative enzyme activity. The enzyme was incubated in buffer at three temperatures, 37°C, 50°C, and 60°C, for up to 12 h to check for its thermal stability, and relative activity was plotted. To enhance the stability of the enzyme, 50% (wt/vol) polyethylene glycol was added, and enzyme activity was checked at 37°C and pH 7.4 (69). Different detergents and organic reagents, such as sodium dodecyl sulfate (SDS), Tween 40, Triton X, ethanol, dimethylformamide (DMF), dimethyl sulfoxide (DMSO), β-mercaptoethanol, and EDTA, at a concentration of 1 mM were used to assess their effect on enzyme activity. Metal ions at a final concentration of 1 mM (K^+^, Mg^2+^, Fe^2+^, Na^+^, Ca^2+^, Cu^2+^, Mn^2+^) were also checked for their effect on enzyme activity. The control used was the enzyme activity at pH 7, 37°C, and without any additive. It was defined as 100% activity.

### Bioinformatic analysis

The gene and protein level similarity of target enzymes was carried out using Clustal Omega Multiple Sequence Alignment (https://www.ebi.ac.uk/Tools/msa/clustalo/). The domain study of the structure of the target protein was carried out using the online tool called InterPro (https://www.ebi.ac.uk/interpro/). The docking of protein thiol peroxidase and ligand asphaltene was carried out using the HDOCK server (70). The 3D structure of thiol peroxidase was predicted using AlphaFold 3 (71), and the ligand structure was constructed using ChemDraw and OpenBabel (72). The protein-ligand interaction was studied on the PLIP online server (73).

### Asphaltene biotransformation

A biotransformation experiment to detect the changes in asphaltene due to the action of enzyme was carried out in which 37.5 mg of asphaltene dissolved in 500 µL of toluene (19) was added to 10 mL of crude lysate containing overexpressed protein in phosphate-buffered saline (PBS) obtained after sonication. An exactly similar methodology was followed to set up a control experiment using an empty pET29a vector transformed in *E. coli* cells and crude lysate subjected to asphaltene. The flasks containing the crude lysate and asphaltene were set at rotating conditions at 200 rpm and 37°C. The flasks were harvested at day 7, day 14, and day 18. The asphaltene fraction of each time point was analyzed using GC-MS.

### Fourier transform infrared spectrophotometry

The extracted asphaltene fraction from both control (crude lysate of induced vector without cloned gene) and test (overexpressed enzyme) was subjected to FTIR to determine any change in the functional groups and bonding. Thermo Fisher Scientific Nicolet iS50 in attenuated total reflectance (ATR) mode of scanning through the wavelength of 400 cm^−1^ to 4,000 cm^−1^ was used.

### Nuclear magnetic resonance

Bruker Advance 800 spectrometer operating at 14.09 T was used for ^1^H- NMR. About 15 mg of extracted asphaltene from both control and test was dissolved in 500 µL of CDCl_3_. The chemical shift obtained at 7.25 ppm was that of CDCl_3_. The spectra were acquired with a pulse angle of 90° (14 μs) and a spectral width of 8.01 kHz with a delay time of 1 s.

### Gas chromatography mass spectrophotometry

The asphaltene extracted from test (incubated with overexpressed enzyme) and control (incubated in PBS buffer) was dissolved in toluene. About 40 µL of the dissolved mixture was further diluted by adding 960 µL of toluene, and it was then filtered using filters with a pore size of 0.2 µm. Agilent Technologies GC-MS 8860 equipped with a HP-5 capillary column was used for the analysis of the asphaltene fraction, and the compounds were detected using Agilent mass detector. The software MassHunter Workstation was used as the database for identification of the compounds. The carrier gas used was helium with a flow rate of 1 mL/min. The temperature of the injector and detector was 300°C and 280°C, respectively. The injection volume used was 5 µL. The oven temperature was set at 65°C for 1 min with a ramp rate of 5°C/min up to 280°C, with a hold time of 15 min.

### Elemental analysis 

Elementar Analysensysteme GmbH, Germany (UNICUBE) was used to analyze the carbon, hydrogen, nitrogen, and sulfur (CHNS) values of treated asphaltene compared to control. Five milligrams of asphaltene was weighed and loaded into a tin capsule, which was then mounted over a carousel to be combusted in a chamber. The combustion was carried out at 1,150°C. The reduction chamber was set at 850°C. The elemental percentage was calculated based on the mass differences obtained.

### Measurement of reduction in aromaticity

Pure asphaltene was dissolved in dichloromethane to a concentration of 2 mg/mL. A reaction mixture containing hexane:isopropanol:100 mM citrate phosphate buffer (pH 7) was made (46:47.7:6.3 vol/vol). To this mixture, 6 µL of asphaltene dissolved in dichloromethane was added. To the test mixture, pure enzyme was added (55). The emission profile was captured at a wavelength ranging from 290 nm to 610 nm at an excitation wavelength of 230 nm. The fluorescence intensity was captured at varying concentrations of enzyme ranging from 5 µg/mL to 20 µg/mL. The enzyme action was measured every 5 min up to a total of 20 min. Similarly, the action of pure enzyme on aromaticity levels of PAHs naphthalene and phenanthrene was studied. About 0.5 mM of compound dissolved in acetonitrile was added to a reaction mixture containing 100 mM potassium phosphate buffer, pH 7.0, 1 mM H_2_O_2_, and 10 µg pure enzyme. The emission profile of naphthalene was captured at an excitation wavelength of 230 nm, whereas for phenanthrene, the excitation profile used was 250 nm. The enzyme turnover number with respect to asphaltene was calculated using the formula (moles of product formed per second) / moles of enzyme used.

### Enzyme action on lignin

Alkali lignin was obtained from Sigma Aldrich, USA. The chemical was dissolved in phosphate buffer to make a stock of 2 mg/mL. To 1 mL of this solution, about 10 µL of purified enzyme was added. The mixture was incubated in shaking conditions at 30°C. The sample was analyzed at time points of 1 h, 2 h, 3 h, 4 h, 5 h, 6 h, and 24 h by measuring absorbance at 280 nm (74, 75).

### Enzyme action on pure PAH compounds

Pure naphthalene and phenanthrene were obtained from Sigma Aldrich, USA. A total reaction mixture of 1 mL was made consisting of 100 mM potassium phosphate buffer, pH 7.0, 1 mM H_2_O_2_, and 10 µg pure enzyme, along with 1 mM of PAH compound dissolved in acetonitrile. Co-solvent solutions of 20% vol/vol and 40% vol/vol were used for naphthalene and phenanthrene, respectively, to give a final concentration of 1 mM in 1 mL reaction mixture. Samples were then analyzed by HPLC (Agilent 1200) with column Agilent Eclipse XDB C18 (4.6 × 150 mm, 5 µm). A mixture of acetonitrile and water (70:30 vol/vol) was used as mobile phase at a flow rate of 0.5 mL/min. The analysis was performed at 30°C. Naphthalene was detected at 220 nm, whereas phenanthrene was detected at 254 nm using a UV diode array detector (DAD) (58, 76).

### Scanning electron microscopy

The treated asphaltene fraction was compared with untreated asphaltene by imaging with SEM. The solid asphaltene sediment was completely dried and then plated with gold. Zeiss EVO 18 SEM instrument was used for imaging.

### Protein action on model oil containing asphaltene

To check the action of thiol peroxidase on asphaltene, a model oil was prepared having 150 mg asphaltene dissolved in 2 mL of toluene followed by the addition of 18 mL hexadecane (19). A lab-scale packed bed column (diameter 4 cm, height 8 cm) was designed in which agarose disks containing immobilized pure thiol peroxidase (77) at a concentration of 2 µg/µL were packed alternately with sand. The agarose disks were formed using 1% agarose in phosphate-buffered saline, to which enzyme was added just before it solidified. To check the stability of the immobilized enzyme, the agarose disk containing immobilized enzyme was incubated in different tubes at room temperature for different time intervals. After incubation, the agarose disk was mashed in an enzyme assay buffer consisting of 50 mM HEPES-NaOH, pH 7.0. About 20 µL was used to check activity of the enzyme. The stability was checked up to 24 h of immobilization in comparison with the control, which was the activity of pure enzyme at pH 7.0, 37°C, without any incubation. About 40 mL of model oil was passed through this column containing a total of 0.6 mg pure protein for 6 h and collected in a beaker. The asphaltene was extracted using n-heptane precipitation, in which the residual asphaltene was ultimately dissolved in toluene. The asphaltene obtained was then dried completely, and its dry weight was recorded. To determine the biotransformation efficiency, the absorbance of the residual asphaltene was measured at 300 nm after dissolving it in toluene, as asphaltene samples used give a maximum absorption at this wavelength (19).

### Protein action on crude oil

Ratawi crude oil with an API gravity of 24.5 was used to check the effect of enzyme on asphaltene content present in natural crude oil. To the packed bed column (diameter 4 cm, height 8 cm) containing 0.6 mg pure protein, about 20 mL crude oil was passed. The total oil was collected after 48 h of flow under the effect of gravity. Asphaltene from the collected fraction as well as the untreated fraction was extracted by n-heptane precipitation. The amount of asphaltene biotransformed with respect to untreated asphaltene was determined by dissolving it in toluene and measuring absorbance at 300 nm.

## SUPPLEMENTAL MATERIAL

Supplemental material aem.00151-25-s0001.pdf Tables S1 to S4; Fig. S1 to S16.

ASM does not own the copyrights to Supplemental Material that may be linked to, or accessed through, an article. The authors have granted ASM a non-exclusive, world-wide license to publish the Supplemental Material files. Please contact the corresponding author directly for reuse.
