# Supplementary material for: Asphaltene biotransformation by a novel enzyme thiol peroxidase from Micrococcus sp. IITD107
Source: Appl Environ Microbiol. 2025 Aug 21;91(9):e00151-25. doi: 10.1128/aem.00151-25 (PMC12442351; doi:10.1128/aem.00151-25)
Supplement: Supplemental material — Tables S1 to S4; Fig. S1 to S16. [file aem.00151-25-s0001.pdf]

Table S1. The various peroxidases found in the genomes of the nine members of the bacterial consortium able to biotransform asphaltene. The enzyme thiol peroxidase is the most abundantly present in the consortium.

S.No	Enzyme	IITD100	IITD101	IITD102	IITD103	IITD104	IIT105	IITD106	IITD107	IITD108
1	NADH peroxidase	Absent	Absent	Absent	Absent	Present	Absent	Absent	Absent	Absent
2	dye decolourising peroxidase	Absent	Absent	Present	Present	Absent	Absent	Absent	Absent	Absent
3	non heme chloroperoxidase	Absent	Absent	Present	Present	Absent	Absent	Absent	Absent	Absent
4	catalase peroxidase	Absent	Absent	Present	Present	Absent	Present	Absent	Absent	Absent
5	thioredoxin peroxidase	Absent	Absent	Present	Present	Absent	Absent	Absent	Absent	Absent
7	non heme dependent bromoperoxidase	Present	Present	Present	Present	Absent	Absent	Absent	Absent	Absent
8	glutathione peroxidase	Absent	Absent	Absent	Absent	Present	Present	Present	Absent	Present
9	thiol peroxidase	Absent	Absent	Absent	Absent	Present	Present	Present	Present	Present

Present

Absent

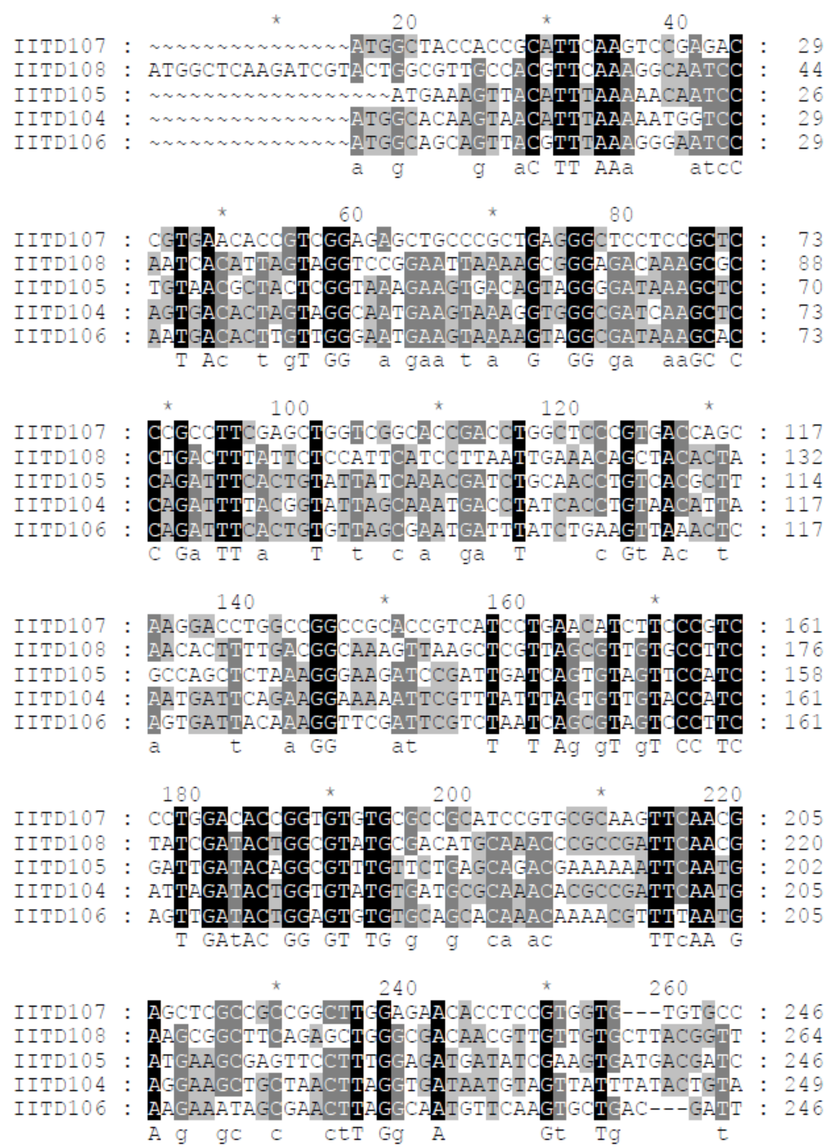

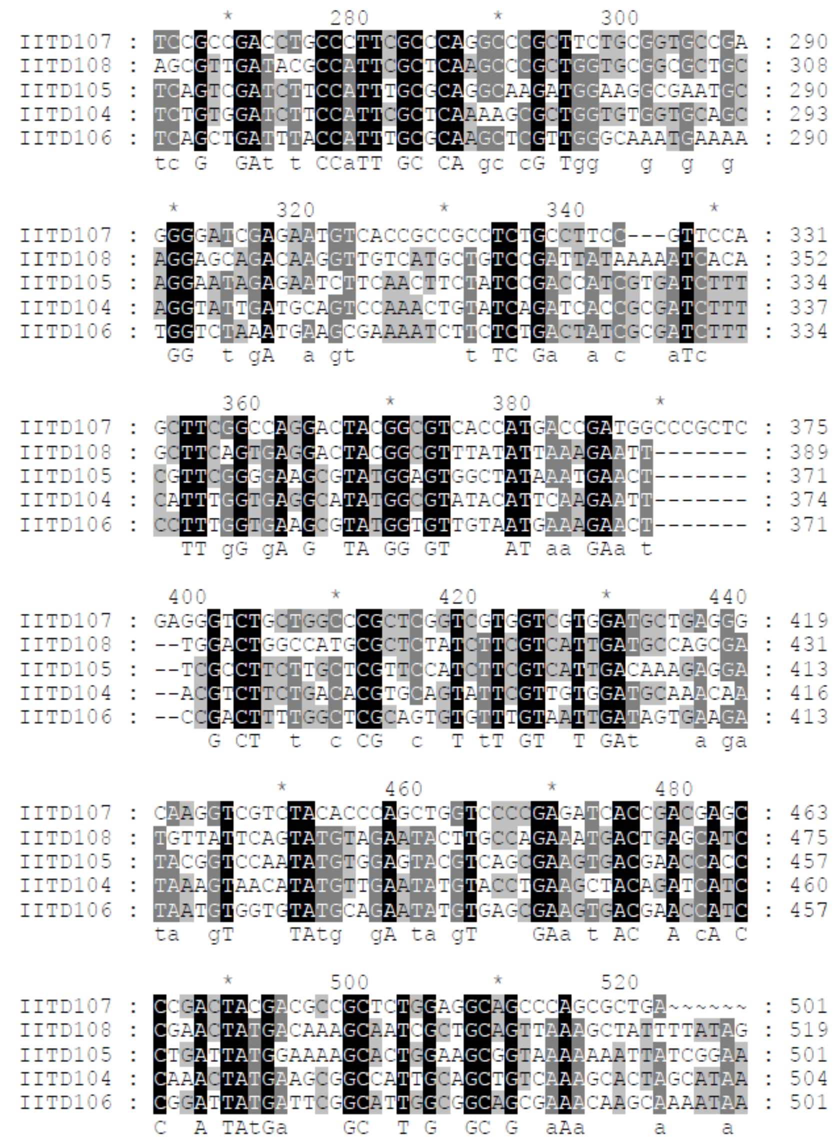

Percent Identity Matrix - created by Clustal2.1

1: IITD107	100.00	46.75	44.99	48.17	46.12
2: IITD108	46.75	100.00	54.49	59.52	54.09
3: IITD105	44.99	54.49	100.00	62.28	63.05
4: IITD104	48.17	59.52	62.28	100.00	64.67
5: IITD106	46.12	54.09	63.05	64.67	100.00

Fig S1. The multiple sequence alignment of gene sequence of *tpx* encoding enzyme thiol peroxidase shows that the sequence from the *Micrococcus* sp. IITD107 is the most unique and shares least identity with other members.

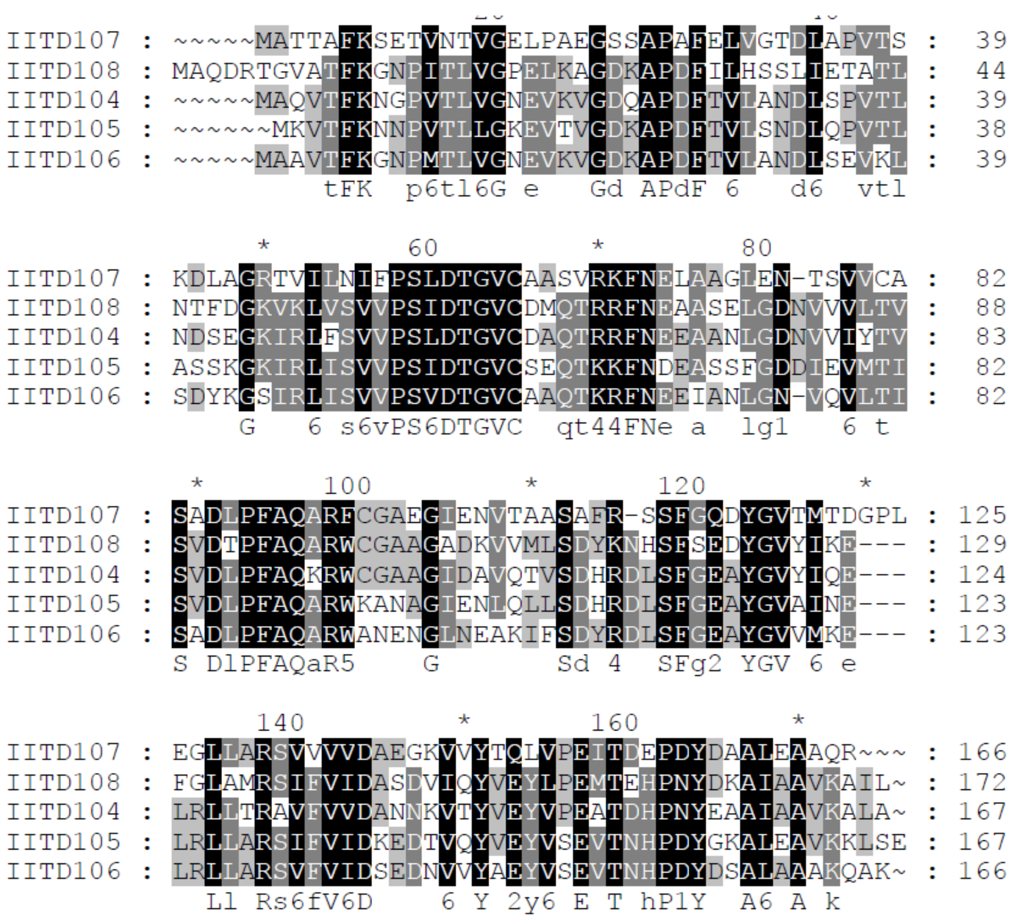

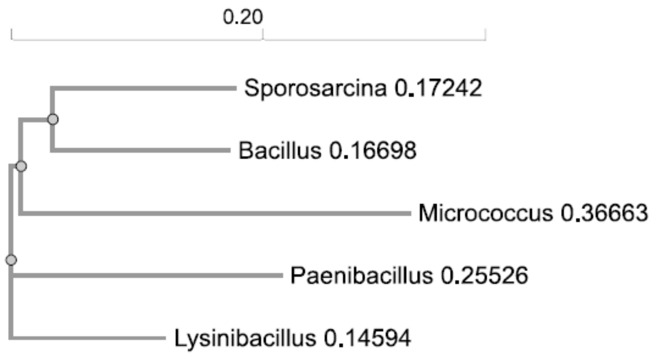

Percent Identity Matrix - created by Clustal2.1

1: IITD107	100.00	37.42	47.24	41.36	45.40
2: IITD108	37.42	100.00	59.88	54.22	51.81
3: IITD104	47.24	59.88	100.00	65.06	65.06
4: IITD105	41.36	54.22	65.06	100.00	66.06
5: IITD106	45.40	51.81	65.06	66.06	100.00

Fig S2. The multiple sequence alignment of protein sequence of enzyme thiol peroxidase between all members of the consortium shows that the sequence from the *Micrococcus* sp. IITD107 is the most unique and shares least identity with others

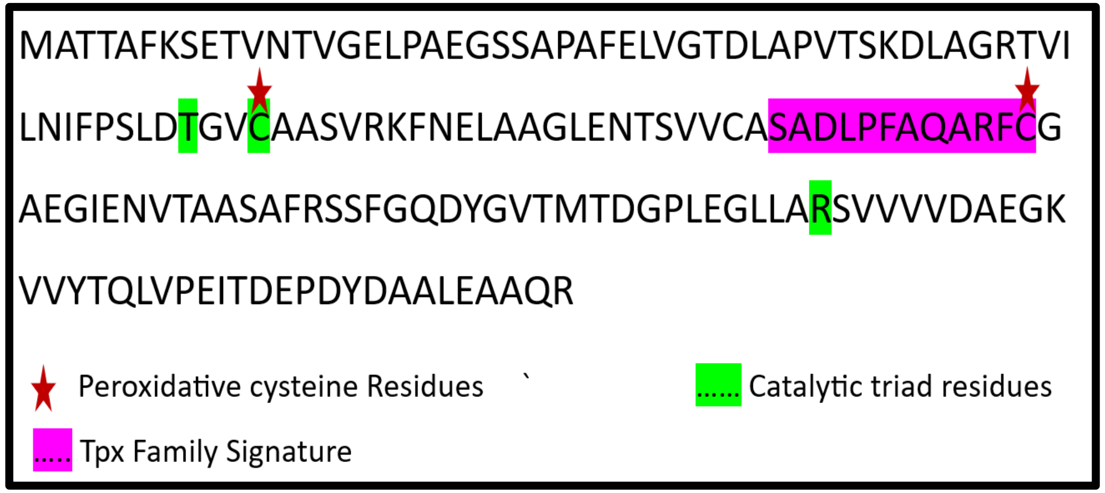

Fig S3. The protein sequence of thiol peroxidase from *Micrococcus* sp. IITD107 with highlighted cysteine residues that play a part in the peroxidative activity of the enzyme. The domain analysis reveals the presence of the thiol peroxidase family signature domain along with conserved residues which are a part of the catalytic triad of the enzyme.

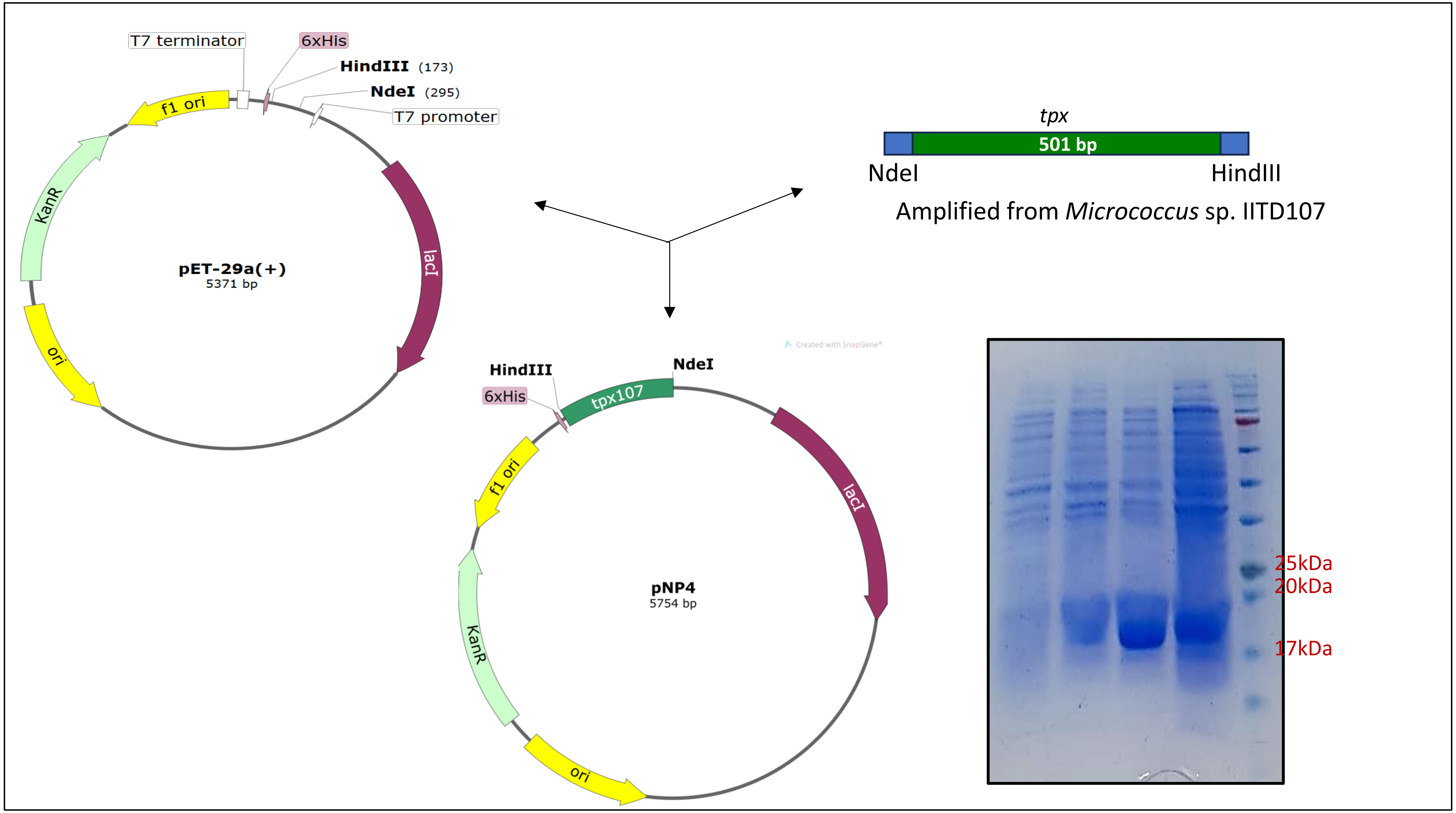

Fig S4. Cloning strategy used in the study using a vector pET29a and cloning of the amplified gene *tpx* from the genome of the *Micrococcus* sp. IITD107 (Accession number -JBHFFN000000000)), in between the sites NdeI and HindIII. The obtained plasmid is pNP4. Protein obtained by gene overexpression is observed at around 18 kDa.

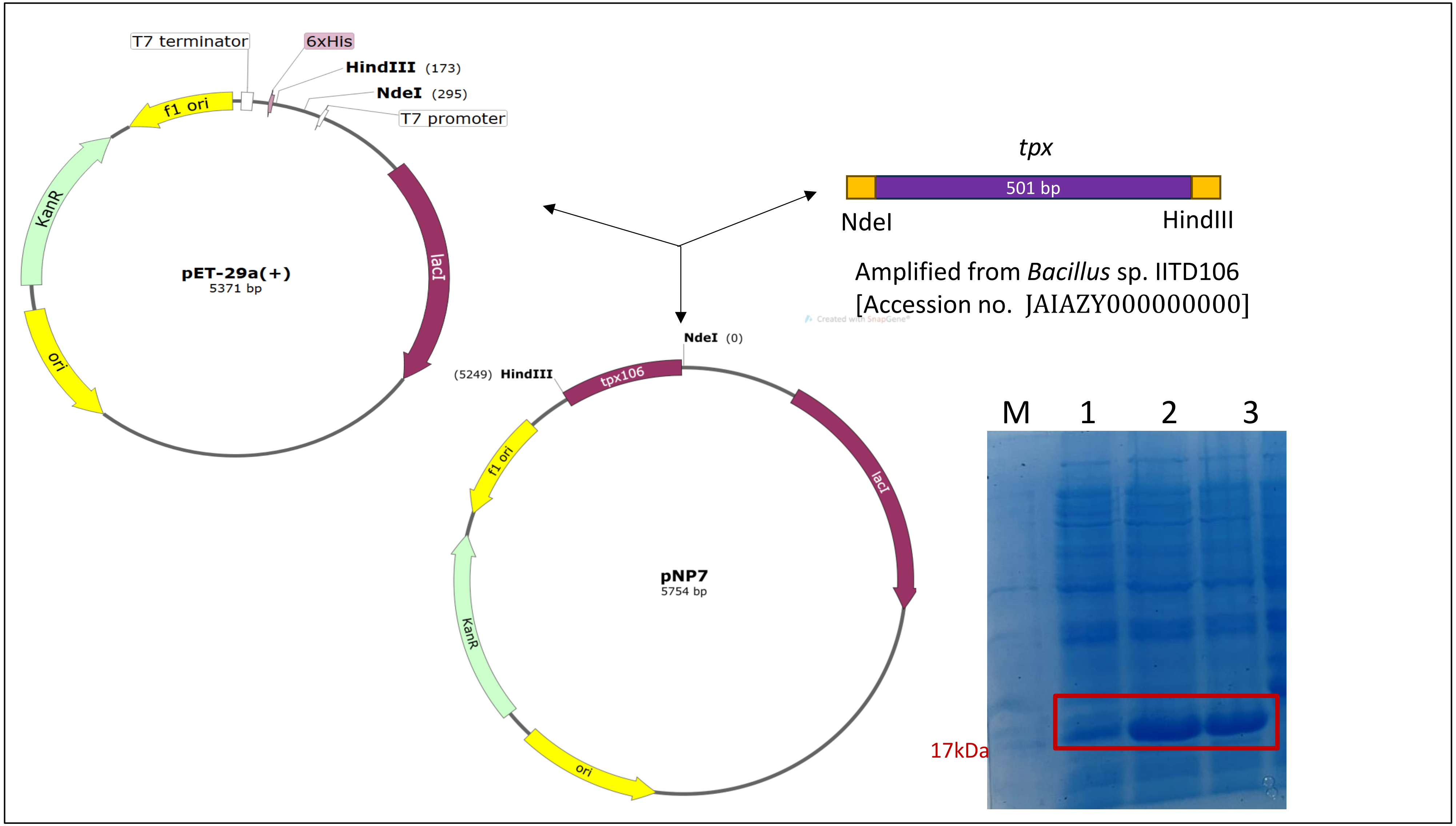

Fig S5. Cloning strategy used in the study using a vector pET29a and cloning of the amplified gene *tpx* from the genome of the *Bacillus* sp. IITD106 (Accession number - JAIAZY000000000), in between the sites NdeI and HindIII. The obtained plasmid is pNP7. Protein obtained by gene overexpression is observed at around 18 kDa.

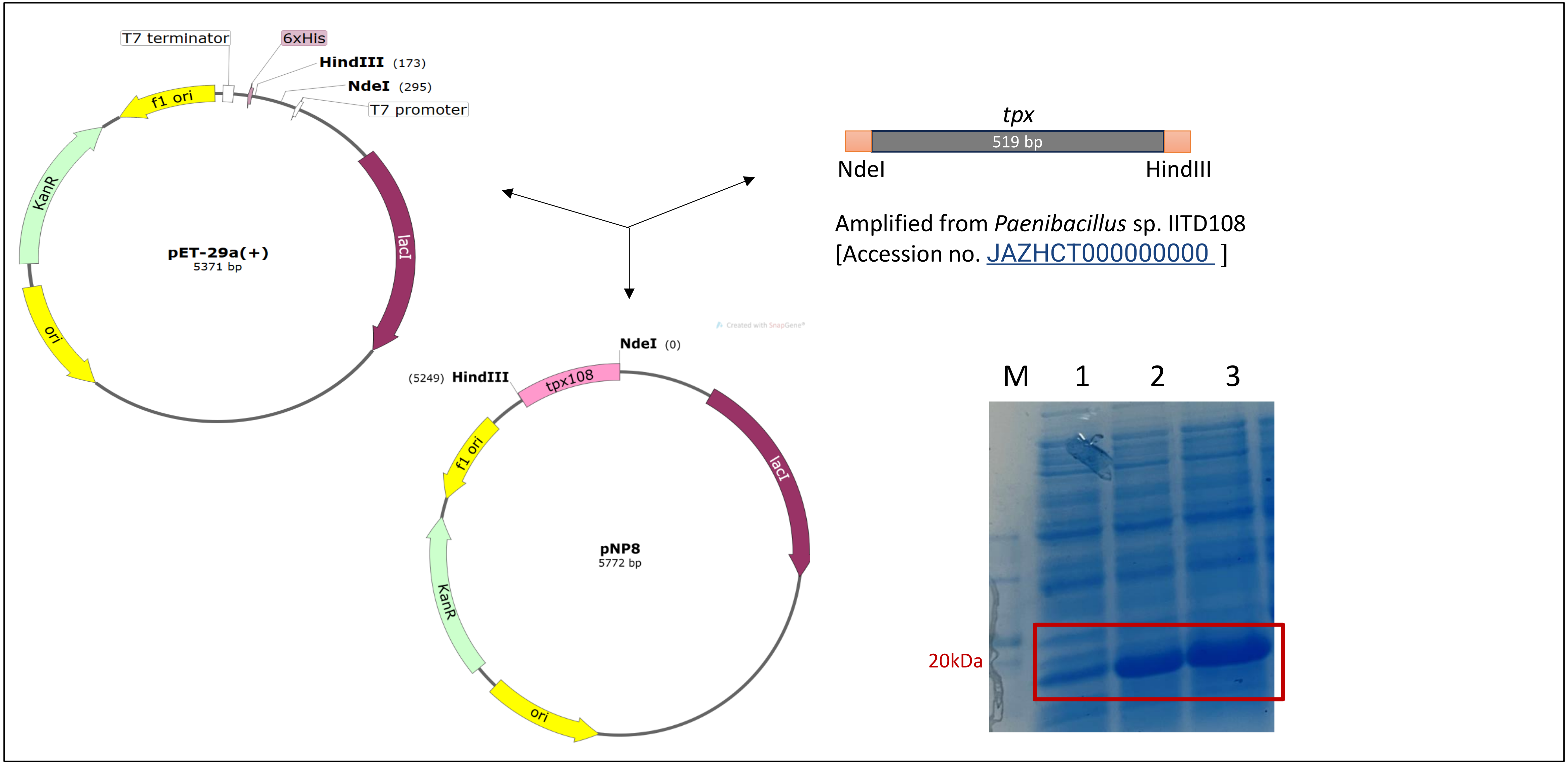

Fig S6. Cloning strategy used in the study using a vector pET29a and cloning of the amplified gene *tpx* from the genome of the *Paenibacillus* sp. IITD108 (Accession number - JAZHCT000000000), in between the sites NdeI and HindIII. The obtained plasmid is pNP8. Protein obtained by gene overexpression is observed at around 18 kDa.

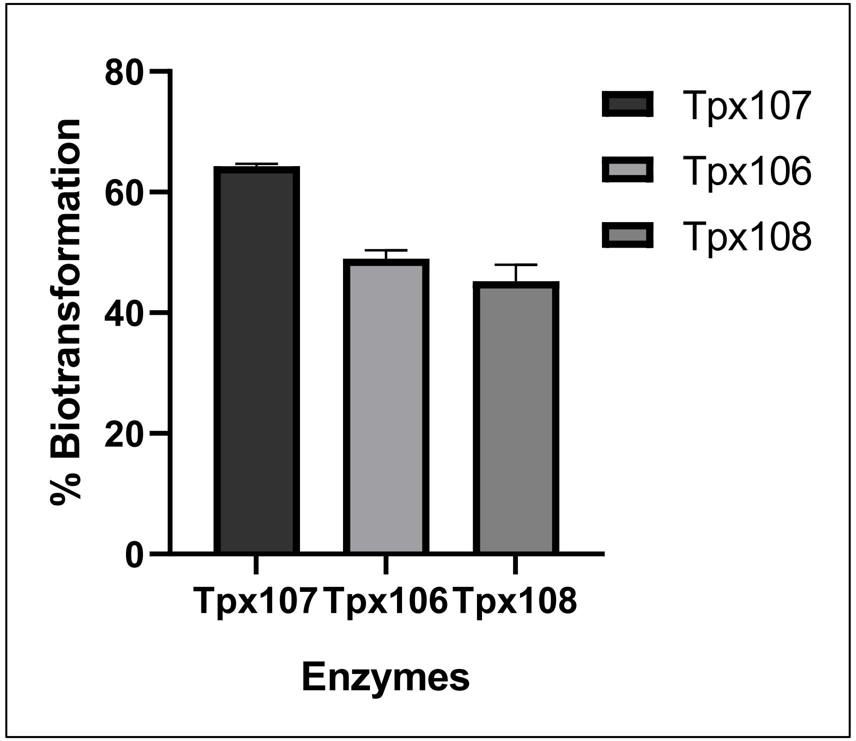

Fig S7. Comparative biotransformation efficiencies of thiol peroxidase from three members of consortium, *Micrococcus* sp. IITD107*, Bacillus* sp. IITD106 *and Paenibacillus* sp. IITD108. The highest biotransformation was achieved by thiol peroxidase from IITD107 of 64.27%.

A.

i.

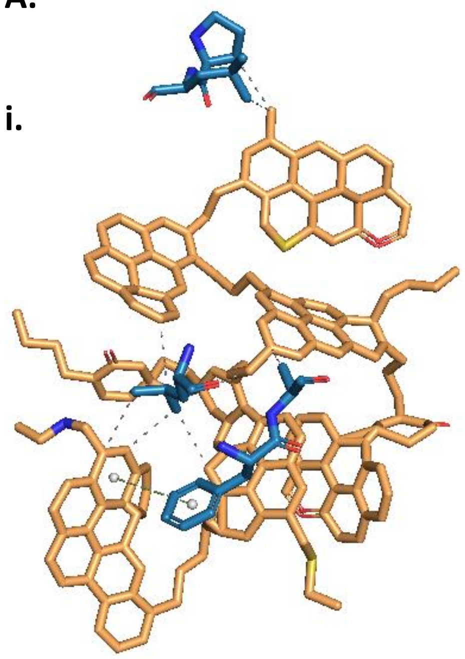

Protein
Ligand
---- Hydrophobic interaction
---- π – stacking

ii

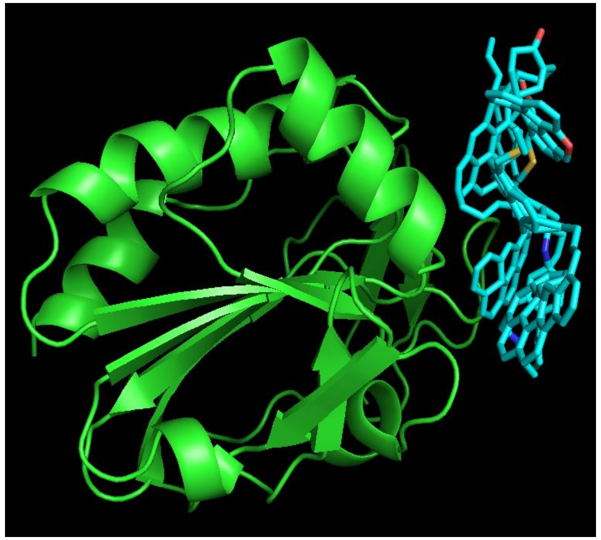

iii

Hydrophobic interaction

Index	Residue	AA	Distance	Ligand Atom	Protein Atom
1	86A	LEU	3.53	1366	610
2	86A	LEU	3.58	1275	609
3	86A	LEU	3.24	1365	609
4	86A	LEU	3.55	1254	607
5	86A	LEU	3.56	1248	608
6	88A	PHE	3.67	1276	625
7	89A	ALA	2.66	1226	633
8	124A	PRO	3.45	1303	885
9	125A	LEU	3.76	1303	895

π – stacking

Index	Residue	AA	Distance	Angle	Offset	Stacking Type	Ligand Atoms
1	88A	PHE	5.48	88.02	0.40	T	1359, 1360, 1364, 1365, 1366, 1367

Fig. S8 A. Interaction between ligand asphaltene and Thiol peroxidase from *Micrococcus* sp. IITD 107.

i. Amino acids interacting with the ligand, ii. Ribbon structure of protein along with interaction with ligand,

ii. Iii. Various types of bonds responsible for interaction.

Fig S8B.

B.

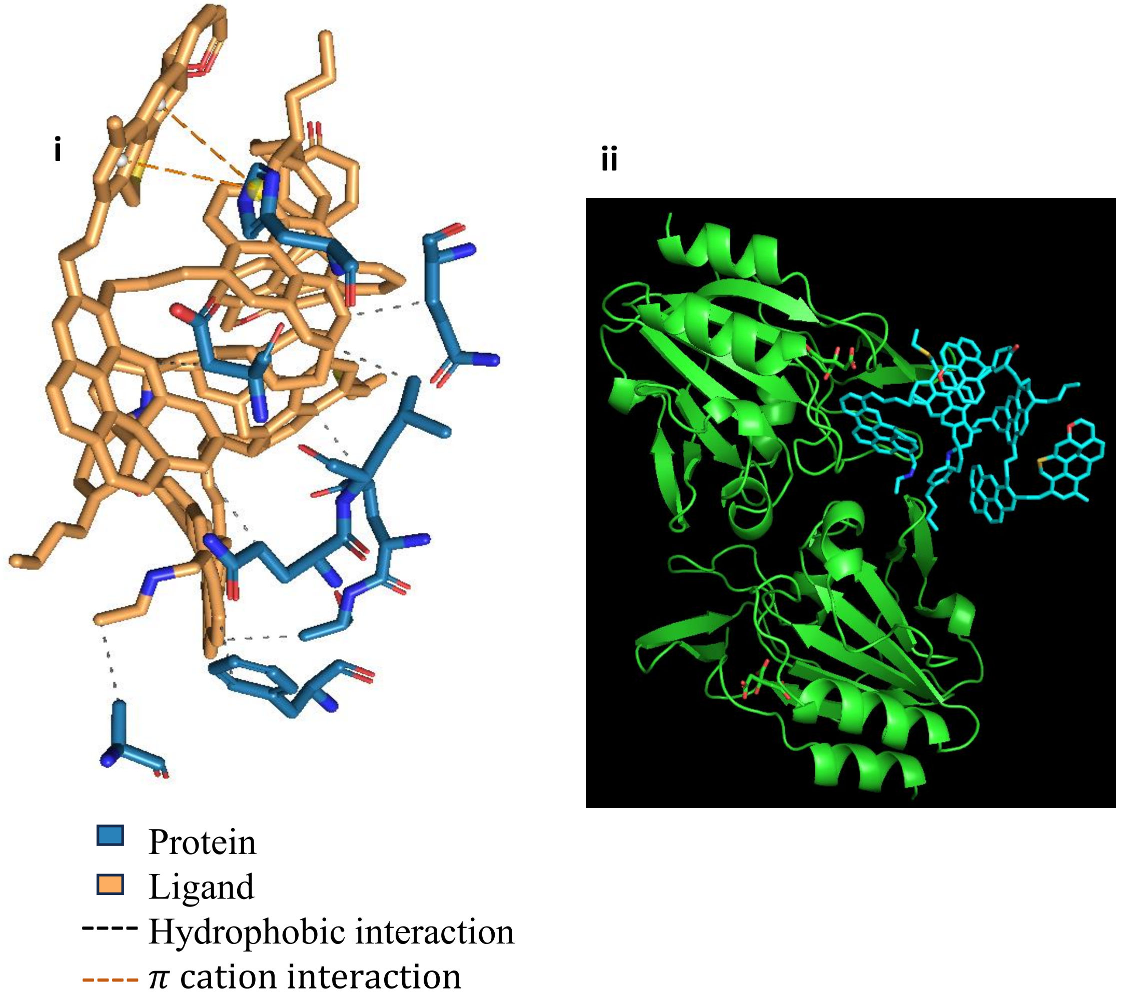

iii.

Hydrophobic interaction

Index	Residue	AA	Distance	Ligand Atom	Protein Atom
1	3B	GLN	3.11	2543	1259
2	20B	GLN	3.68	2598	1387
3	21B	ALA	3.63	2673	1395
4	146B	PHE	3.14	2684	2325
5	148B	GLN	3.69	2563	2340
6	149B	LEU	3.50	2542	2351
7	151B	ASP	3.38	2559	2363
8	161B	ALA	3.74	2693	2447

π cation interaction

Index	Residue	AA	Distance	Offset	Protein charged?	Ligand Group	Ligand Atoms
1	6B	HIS	4.61	1.64	✔	Aromatic	2621, 2622, 2626, 2627, 2628, 2629
2	6B	HIS	4.50	0.80	✔	Aromatic	2616, 2617, 2618, 2620, 2621, 2622

Fig S8 B. Interaction between ligand asphaltene and Thiol peroxidase from *E.coli* (3HVS).
i. Amino acids interacting with the ligand, ii. Ribbon structure of protein along with interaction with ligand, iii. Various types of bonds responsible for interaction.

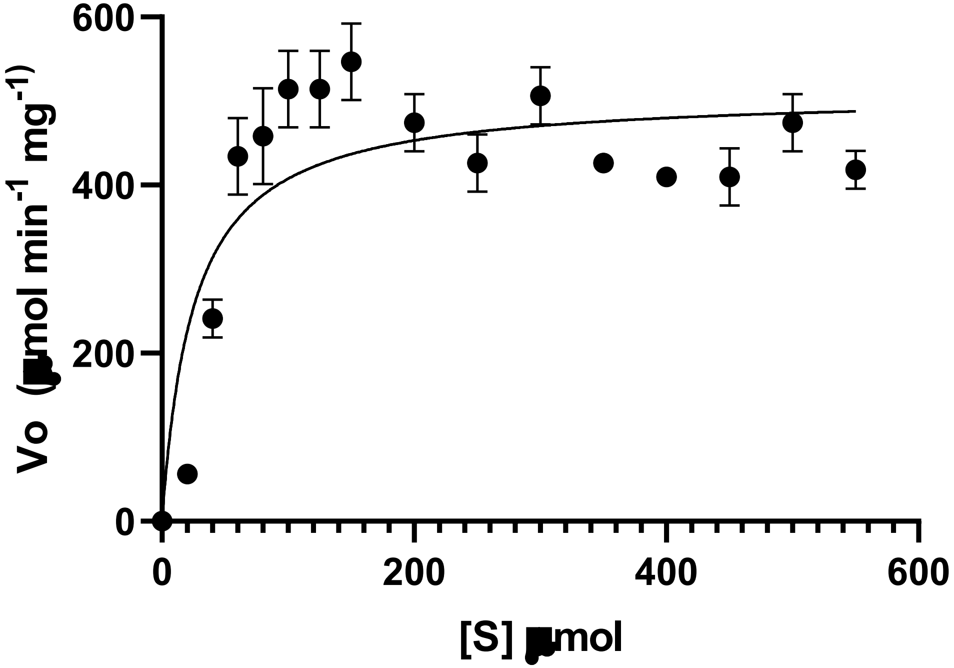

Fig. S9 Michaelis Menten plot for enzyme thiol peroxidase. The K_m is 25.14 µM and V_{max} is 510 µM/min.mg protein. The 95% confidence interval for V_{max} was 460 to 565.7 and for K_m was 13.2 to 42.55. Error bars represent standard deviation between duplicate set of samples.

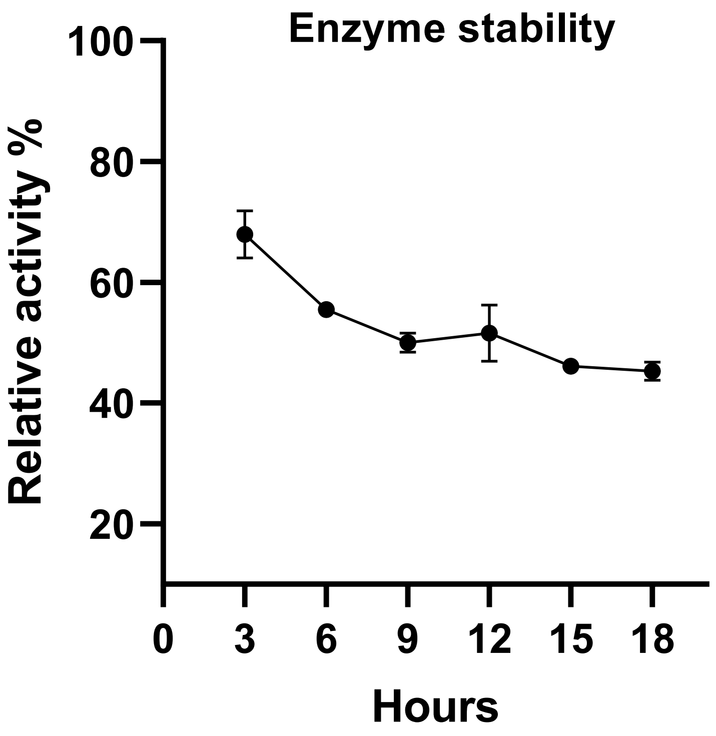

Fig S10. The stability of purified enzyme improved after addition of PEG. About 10µl of PEG4000 was added to 0.5 ml reaction mixture containing 10ug of protein. The enzyme activity was calculated at 37°C every 3 hour incubation, up to a total of 18 hours.

Control Asphaltene

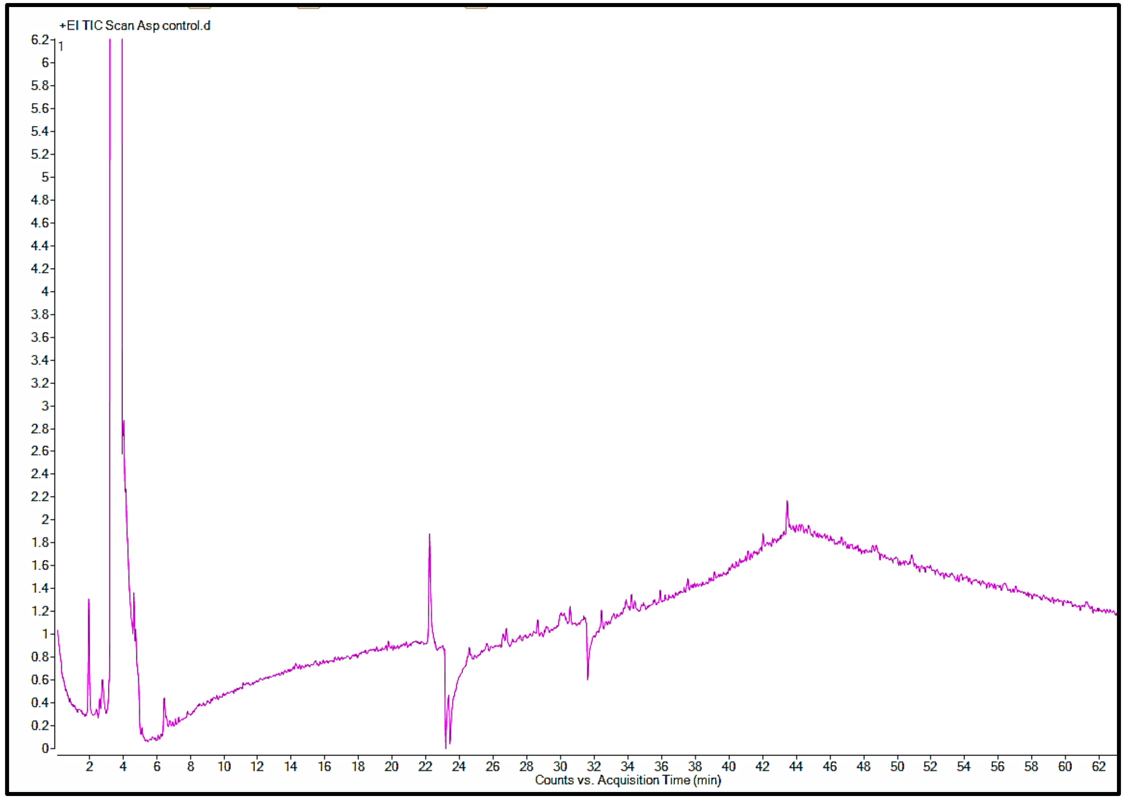

Fig S11 A. The GC MS chromatogram of control sample of asphaltene untreated and raw at day 0. The sample was prepared by dissolving asphaltene in toluene.

Control Day 18

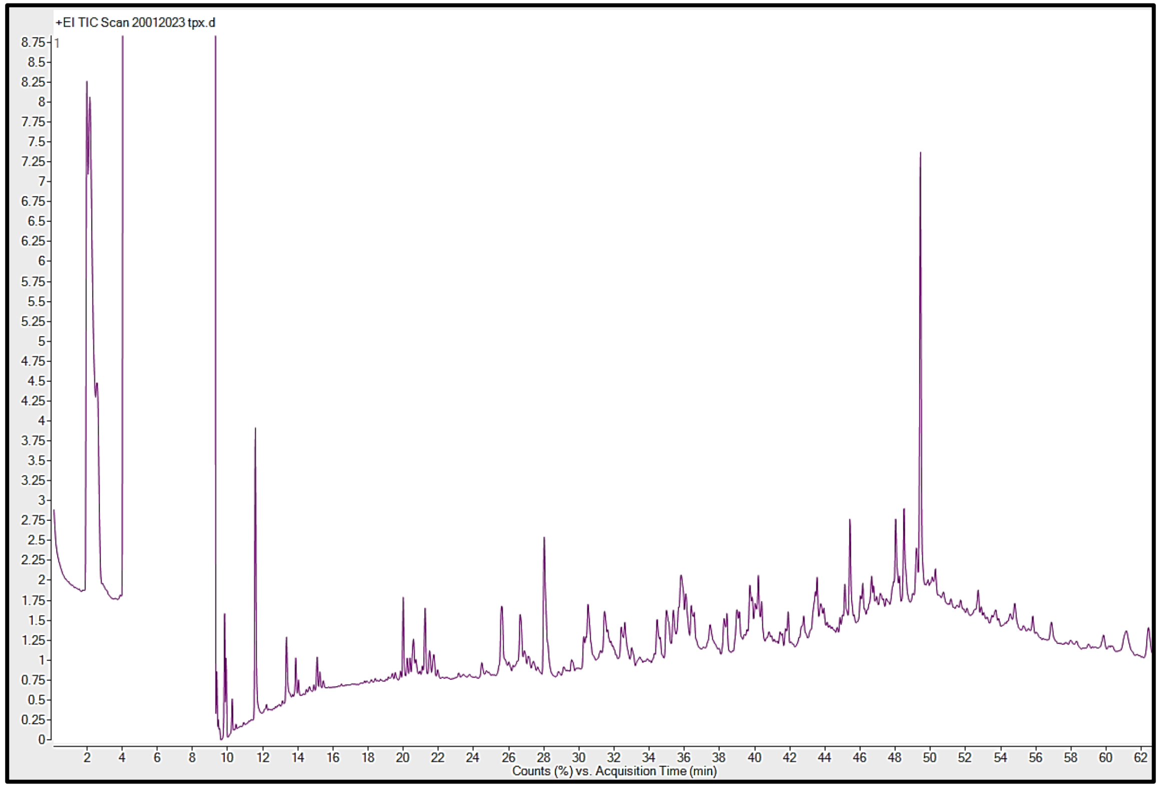

Fig S11 B. The GC MS chromatogram of control sample of asphaltene post 18 days of incubation. The crude lysate of wild type *E.coli* cells without the vector was used to treat asphaltene.

S.No	Compounds	Retention Time	Control	Day 7	Day 14	Day 18
1	Ethylbenzene	10.07	++++	+++	++	+
2	Benzene, [3-(2-cyclohexylethyl)-6-cyclopentylhexyl]-	10.14	-	++++	++	+
3	1,3- cyclopentadiene, 5-(1-methylethylidene)	10.46	++++	+++	++	+
4	p-Xylene	10.52	++++	+++	++	+
5	Benzene, 1,3-dimethyl-	10.95	++++	+++	++	++
6	benzene 3 pentenyl	11.65	++++	+++	++	+
7	Benzaldehyde	11.71	++++	+++	+++	++
8	benzene butane nitrile	12.85	++++	+++	+++	+++
9	Benzyl alcohol	13.63	++++	+++	+++	++
10	1- pentanol, 5 (phenylmethoxy)	13.98	++++	+++	+++	++
11	2,4-Difluorobenzene, 1-benzyloxy-	14.14	++++	+++	++	+
12	Octadecane, 6-methyl-	14.16	++++	+++	++	+
13	Decane, 2,4,6-trimethyl-	15.25	++++	+++	++	+
14	Benzeneacetic acid, 2-tridecyl ester	15.26	++++	+++	++	+
15	Benzenepropanoic acid, α-(hydroxyamino)-	20.51	++++	+++	++	-
16	Dodecane, 2,6,10- trimethyl-	21.25	++++	+++	++	+
18	3-Bromo-4-methyl-2,3-dihydro-thiophene 1,1-dioxide	26.77	-	++++	+++	+++
19	Heptadecane	27.99	++++	++	++	+
20	Hexadecane	28.01	++++	++	++	+
21	Pentadecane	28.02	++++	++	++	+
22	Heptadecane, 2,6,10,14-tetramethyl-	28.03,	++++	++	++	+
23	Nonadecane	28.05	++++	++	++	+
24	Benzothiazole, 2-(2-hydroxyethylthio)	35.39	-	++++	+++	++
25	Hexadecanoic acid	35.7	++++	+++	+++	++
26	Tetradecane, 2,6,10- trimethyl-	35.82	++++	+	+	+
27	Octadecanesulphonyl chloride	37.8	++++	+	+	+
28	Pentacosane	38.23	++++	+	+	+
29	2-Methoxymyristic acid	40.42	++++	++	++	-
30	Phenol, 4,4'-(1-methylethylidene)bis-	40.44	++++	++	++	-
31	Hentriacontane	43.81	++++	+	+	+
32	Hexadecanoic acid, 2-hydroxy-1-(hydroxymethyl)ethyl ester	45.18	++++	+++	+++	+++
33	4-methyldocosane	46.06	++++	++	++	+
34	Dodecanoic acid, 3-hydroxy	46.41	++++	++	++	+
35	Tetracontane	47.15	++++	+	+	+
36	Octadecanoic acid, 2-hydroxy-1-(hydroxymethyl)ethyl ester	48.53	++++	+++	+++	++
37	Octadecanal, 2-bromo-	49.22	++++	+++	+++	+++
38	13-Docosenamide, (Z)-	49.46,	++++	++++	++++	+++
39	Octadecane, 3-ethyl-5-(2-ethylbutyl)-	49.69	++++	+++	++	++
40	Octadecanoic acid, 2-propenyl ester	50.88	++++	+	+	-

Table S2. The various metabolites detected in GC MS along with comparison in peak length with respect to control.
++++ maximum area
+++ 0-30% reduction in area
++ 30-60% reduction in area
+ 60-95% reduction in area

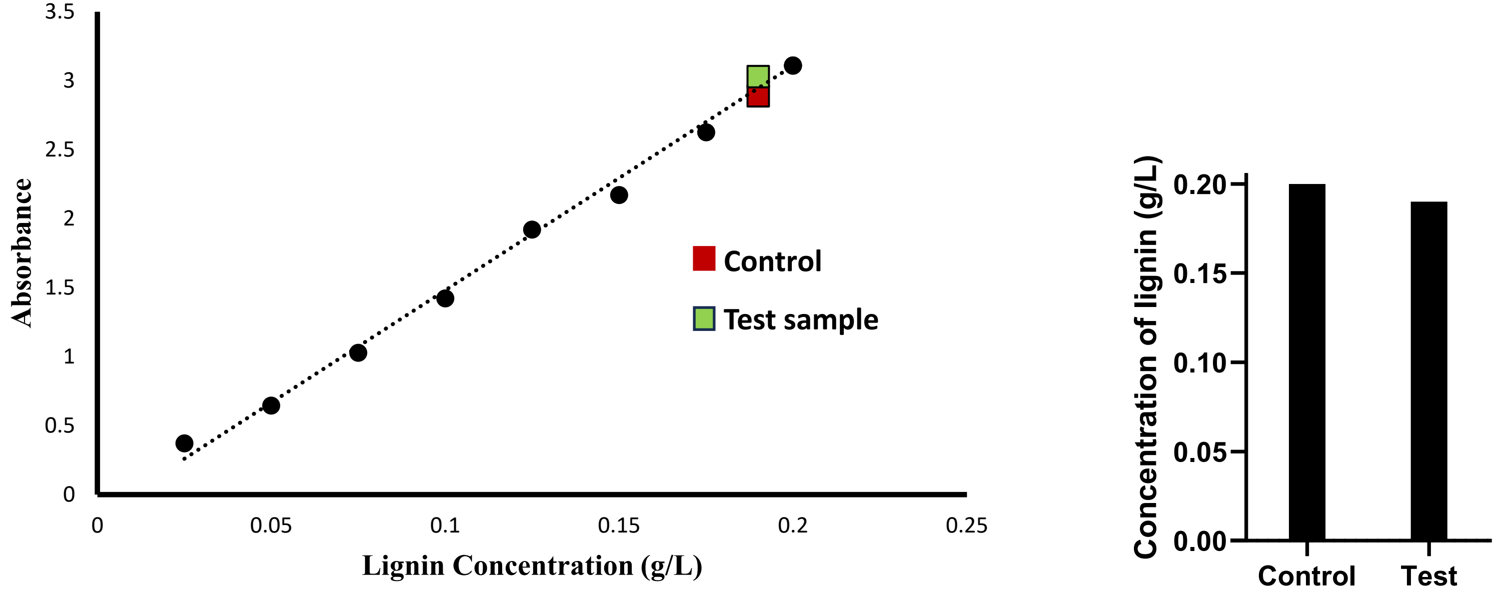

Fig S12. The action of purified enzyme on lignin was tested and no change in lignin was observed even after 24 hrs of incubation with enzyme. Lignin was incubated with purified enzyme at 30°C for 24 h and measured by absorbance at 280 nm. The red box indicates the control sample measured prior to incubation, and the green box indicates the sample after incubation with the purified enzyme.

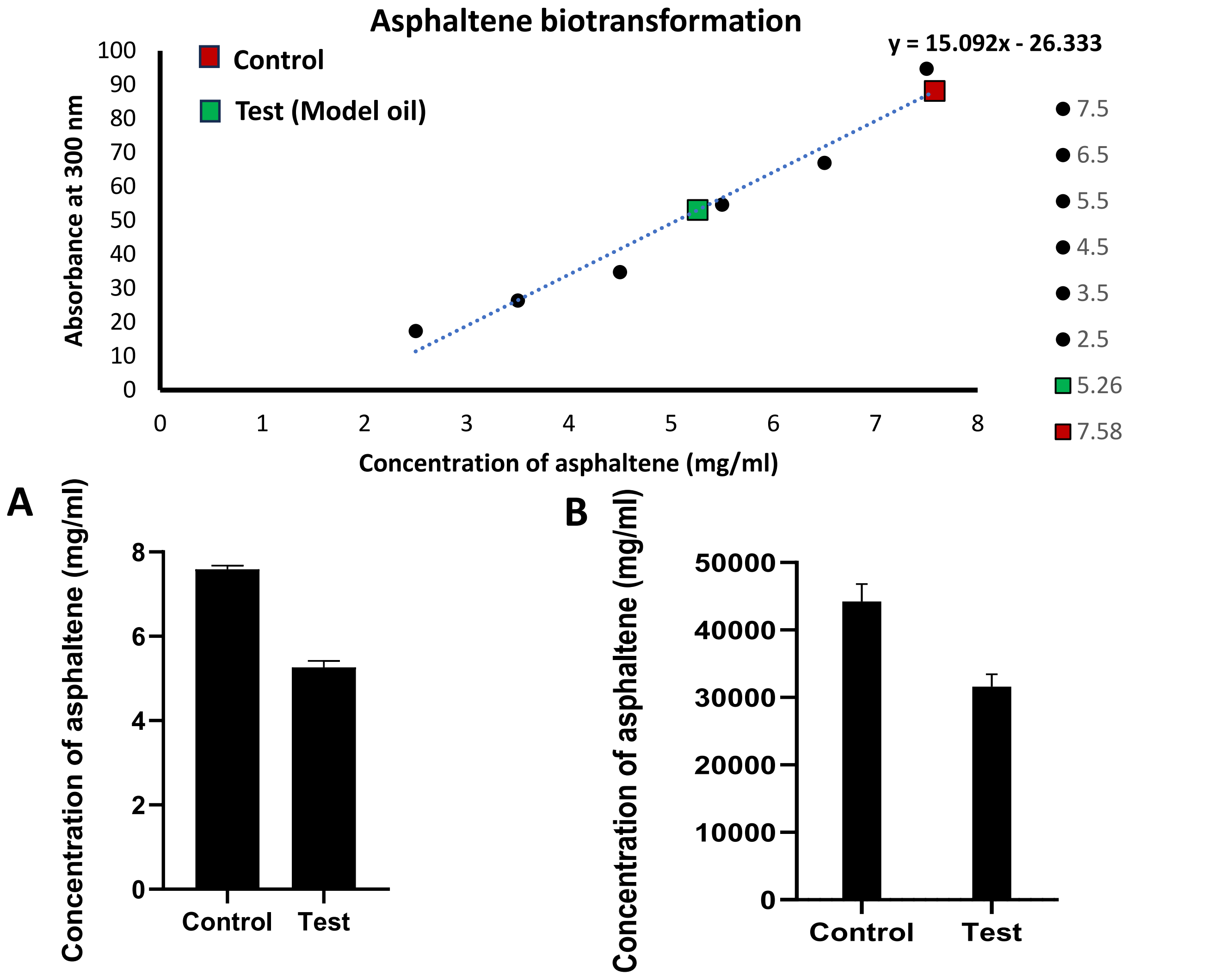

Asphaltene extracted after biotransformation of model oil with and without immobilized enzyme

Asphaltene extracted after biotransformation of crude oil with and without immobilized enzyme

Fig. S13 A The asphaltene biotransformation of treated and untreated asphaltene fraction from model oil with purified and immobilized enzyme showed the asphaltene biotransformation of 30%. By spectrophotometric method, where absorbance was recorded at 300 nm.

B. The extracted asphaltene from crude oil was found to attain 28% biotransformation with respect to control. The concentration of asphaltene before and after treatment was determined from the standard curve equation.

Table S3. Biotransformation of asphaltene present in oil indicating a decrease in its weight by immobilized enzyme.

S.No	Fraction of asphaltene	Weight of asphaltene obtained
1	Untreated (Model Oil)	260 mg
2	Treated (Model Oil)	170 mg
3	Change in weight (Model Oil)	34%
4	Untreated (crude oil)	14.76 gm
5	Treated (crude oil)	11.28 gm
6	Change in weight (crude oil)	23.57%

A.

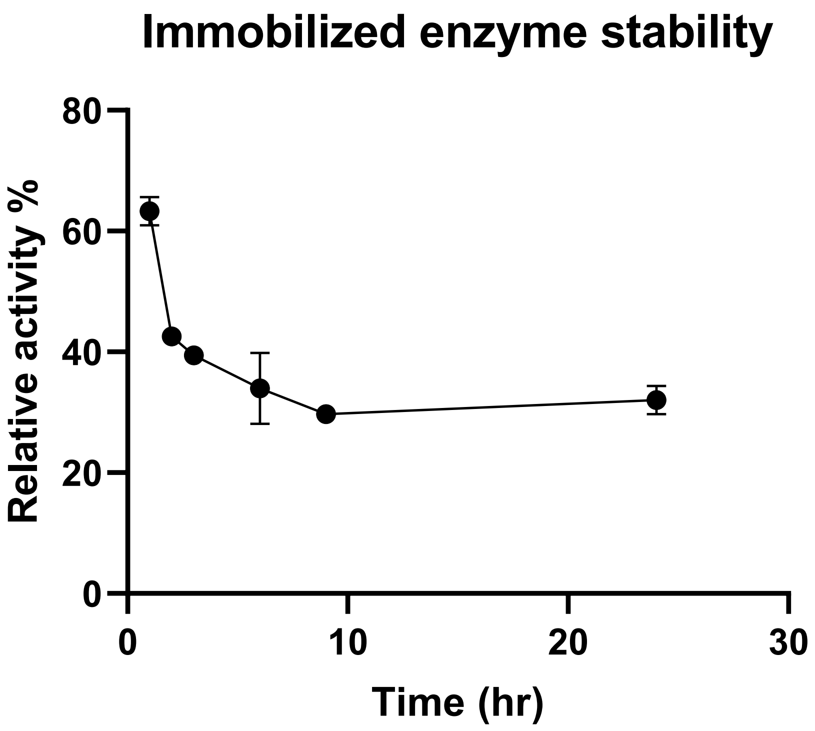

Fig S14 A. The immobilized enzyme was checked for its stability and it was found to retain about 32% activity after 24 hrs of immobilization.

B.

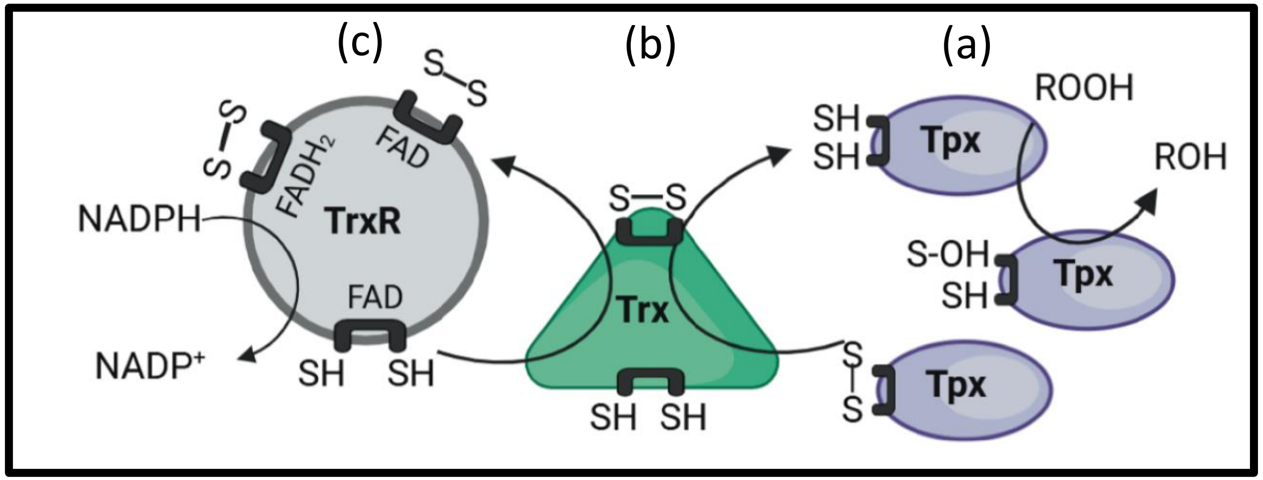

Fig. S14 B. The general mechanism of thiol peroxidase shows the formation of disulfide bond for catalytic conversion of peroxides. The disulfide bridge is resolved with the help of Thioredoxin (Trx) which in turn requires the enzyme Thioredoxin reductase (TrxR). (a) In case of oxidative stress, the peroxidative cysteine gets oxidized to sulfenic acid and then with the help of the resolving cysteine, a disulfide bond is formed with the release of hydroxyl group. (b) This disulfide bond is then resolved with the help of Trx and the reduced enzyme is regenerated. (c) The disulfide bond generated in Trx is then relieved by the action of TrxR, by utilizing NADPH as hydrogen donor.

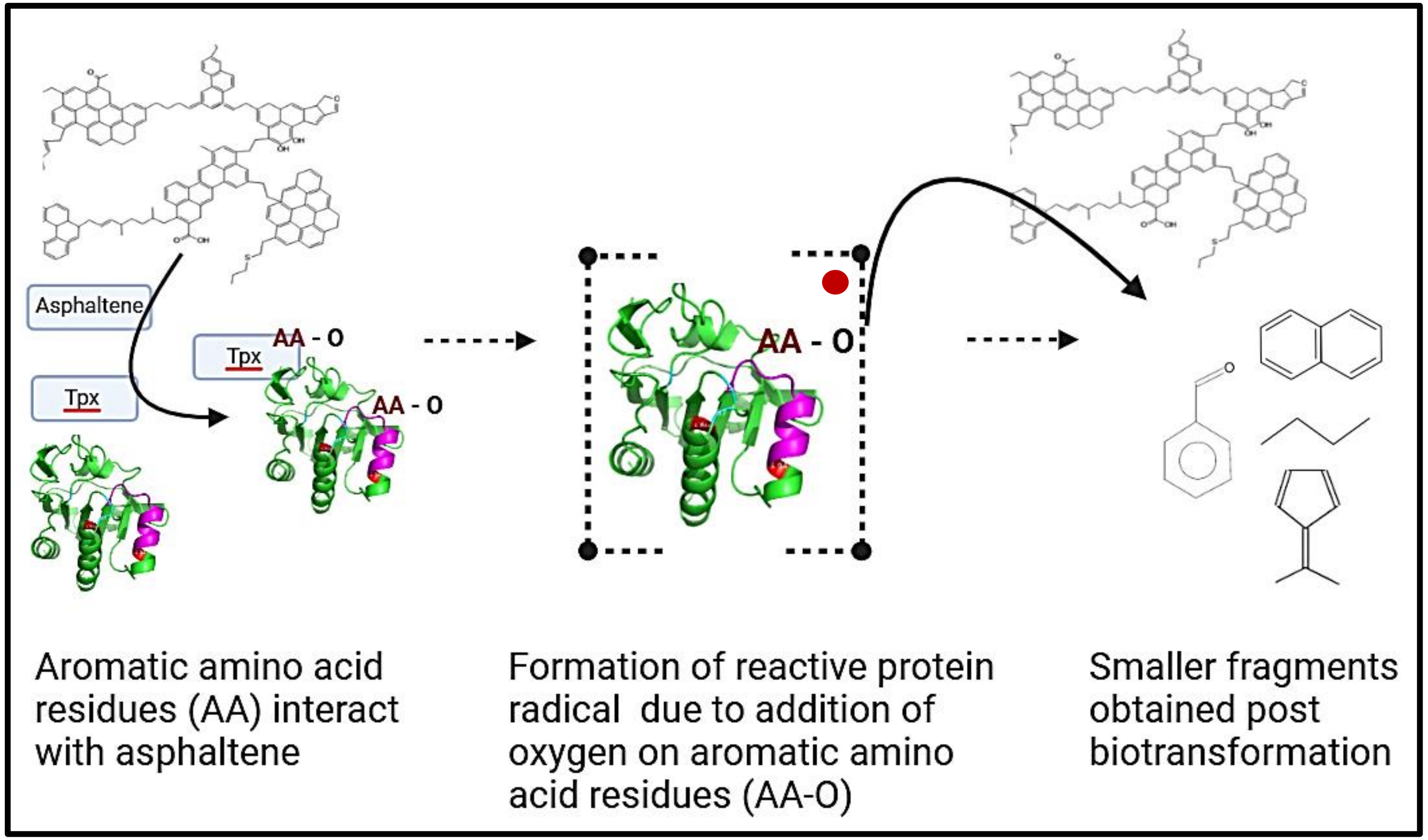

Fig. S15 Hypothetical interaction of protein with asphaltene leading to formation of protein radical due to oxidation of aromatic amino acid residues leading to asphaltene biotransformation

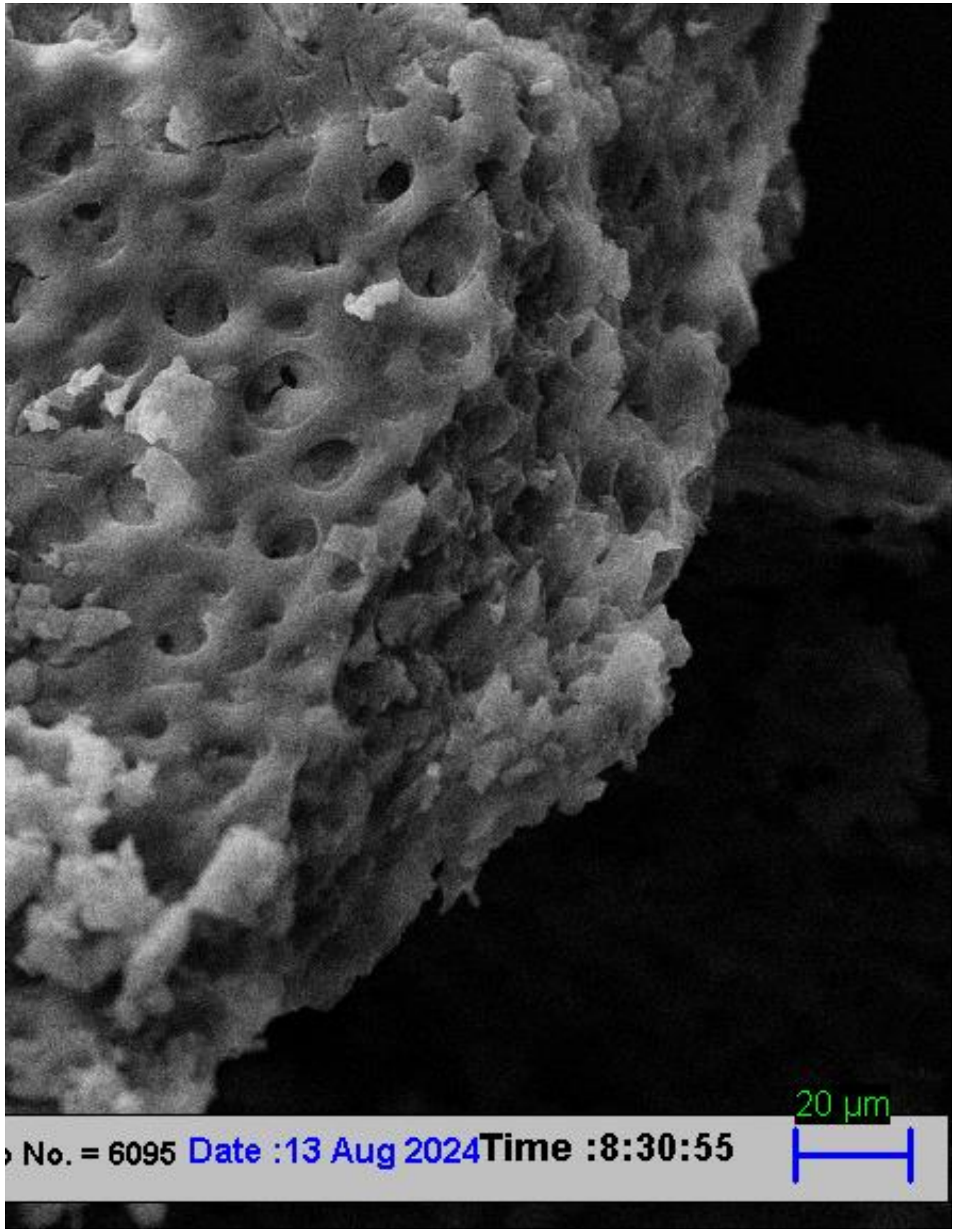

Fig S16. Magnified view of asphaltene treated with thiol peroxidase expressing crude cell lysate revealing porous carbon structure

Table S4. Primers used for cloning of thiol peroxidase from genome of *Bacillus* sp. IITD106, *Micrococcus* sp. IITD107 and *Paenibacillus* sp. IITD108

S.No	Primers	Sequence
1	IITD106 FP	5’ GACT *CATATG* ATGGCAGCAGTTACGTTTAAAGGGAATC 3’
2	IITD106 RP	5’ GATC *AAGCTT* TTTTGCTTGTTTCGCTGCCGCCAATGC 3’
3	IITD107 FP	5’AATC *CATAT G*ATGGCTACCACCGCATTCAAGTC3’
4	IITD107 RP	5’ AATA *AAGCTT*GCGCTGGGCTGCCTCCAGAGCG3’
5	IITD108 FP	5’ GACT *CATATG* ATGGCTCAAGATCGTACTGGCGTTGCCAC 3’
6	IITD108 RP	5’ ATCG *AAGCTT* TAAAATAGCTTTAACTGCAGCGATTGCT 3’
